# Crystalline Divinyldiarsene Radical Cations and Dications

**DOI:** 10.1002/anie.201909144

**Published:** 2019-10-23

**Authors:** Mahendra K. Sharma, Sebastian Blomeyer, Beate Neumann, Hans‐Georg Stammler, Maurice van Gastel, Alexander Hinz, Rajendra S. Ghadwal

**Affiliations:** ^1^ Anorganische Molekülchemie und Katalyse Lehrstuhl für Anorganische Chemie und Strukturchemie Centrum für Molekulare Materialien Fakultät für Chemie Universität Bielefeld Universitätsstr. 25 33615 Bielefeld Germany; ^2^ Max-Planck-Institut für Kohlenforschung Molecular Theory and Spectroscopy Kaiser-Wilhelm-Platz 1 Mülheim an der Ruhr 45470 Germany; ^3^ Institute of Inorganic Chemistry Karlsruhe Institute of Technology (KIT) Engesserstr. 15 76131 Karlsruhe Germany

**Keywords:** arsenic, dications, main-group compounds, π ligands, radicals

## Abstract

The divinyldiarsene radical cations [{(NHC)C(Ph)}As]_2_(GaCl_4_) (NHC=IPr: C{(NDipp)CH}_2_ **3**; SIPr: C{(NDipp)CH_2_}_2_ **4**; Dipp=2,6‐*i*Pr_2_C_6_H_3_) and dications [{(NHC)C(Ph)}As]_2_(GaCl_4_)_2_ (NHC=IPr **5**; SIPr **6**) are readily accessible as crystalline solids on sequential one‐electron oxidation of the corresponding divinyldiarsenes [{(NHC)C(Ph)}As]_2_ (NHC=IPr **1**; SIPr **2**) with GaCl_3_. Compounds **3**–**6** have been characterized by X‐ray diffraction, cyclic voltammetry, EPR/NMR spectroscopy, and UV/vis absorption spectroscopy as well as DFT calculations. The sequential removal of one electron from the HOMO, that is mainly the As−As π‐bond, of **1** and **2** leads to successive elongation of the As=As bond and contraction of the C−As bonds from **1**/**2**→**3**/**4**→**5**/**6**. The UV/vis spectrum of **3** and **4** each exhibits a strong absorption in the visible region associated with SOMO‐related transitions. The EPR spectrum of **3** and **4** each shows a broadened septet owing to coupling of the unpaired electron with two ^75^As (*I*=3/2) nuclei.

## Graphical abstract

**One‐by‐one** electron oxidation of diarsenes [As_2_] featuring very efficient π‐donor N‐heterocyclic vinyl substituents with GaCl_3_ leads to the formation of radical cations [As_2_]^+.^ and dications [As]^2+^ as crystalline solids. Experimental and computational studies revealed the delocalization of unpaired electron over the π‐conjugated CAs_2_C framework.

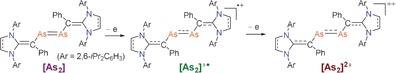

Stable radicals are appealing synthetic targets in main‐group chemistry^1^ because they challenge conventional bonding paradigms as well as exhibit intriguing electronic structure and physical properties. Among the heavier Group 15 elements (P, As, Sb, Bi), numerous phosphorus‐centered stable radicals^2^ have been isolated and structurally characterized. However, the number of crystallographically characterized arsenic,2g, 2k, 2p, 3 antimony,^4^ and bismuth^5^ radicals remained limited.

In 2013, Robinson et al. reported the first stable arsenic radical cation **I** (Figure 1)3a by one‐electron oxidation of an N‐heterocyclic carbene (NHC)‐stabilized diatomic arsenic compound (IPr)_2_As_2_.^6^ Grützmacher and co‐workers reported the neutral radical **II** containing NHC‐phosphinidene substituents.2k Schulz et al. isolated singlet diradicaloids **III**‐E (E=P or As) featuring a 6π‐electron four‐membered N_2_E_2_ ring with a considerable open‐shell character.2g, 3b Consequently, **III**‐E undergo one‐electron oxidation to afford the 5π‐electron radical cations **IV**‐E.2p Very recently, Wang and co‐workers reported the radical cations Ar_3_As^.+^ (**V**) (Ar=*i*Pr_3_C_6_H_2_ or *i*Pr_2_C_6_H_3_).^7^ The high‐lying HOMO of **III**‐E and Ar_3_As facilitates one‐electron oxidation giving rise to radical cations **IV**‐E and **V**, respectively. We recently reported crystalline divinyldiarsenes **1** and **2** derived from classical NHCs, which exhibit remarkably small HOMO–LUMO energy gap of 3.86 eV and 4.24 eV, respectively.^8^ The high‐lying HOMO of **1** (−4.42 eV) and **2** (−5.28 eV) encouraged us to probe the synthetic viability of corresponding stable radical cations on one‐electron oxidation of **1** and **2**. Herein, we report the synthesis of crystalline divinyldiarsene radical cations [{(NHC)C(Ph)}As]_2_(GaCl_4_) (NHC=IPr: C{(NDipp)CH}_2_ **3**; SIPr: C{(NDipp)CH_2_}_2_ **4**; Dipp=2,6‐*i*Pr_2_C_6_H_3_) as well as dications [{(NHC)C(Ph)}As]_2_(GaCl_4_)_2_ (NHC=IPr **5**; SIPr **6**).

**Figure 1:**
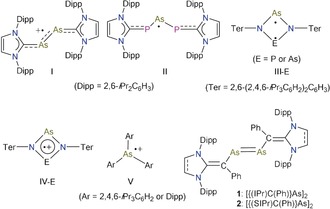
Structurally characterized arsenic‐centered radicals **I**–**V** and divinyldiarsenes **1** and **2**.

We commenced our studies with electrochemical analyses of **1** and **2** (see the Supporting Information). The cyclic voltammograms (CVs) of **1** and **2** exhibit two one‐electron redox events (**1**: −1.00, −0.67; **2**: −0.92, −0.51 V), which may be tentatively assigned to the related radical cations (**1** or **2**)^.+^ and dications (**1** or **2**)^2+^, respectively (Supporting Information, Figure F1 and Table T5). One additional wave at −1.28 V was observed for **1** and is most likely associated with the reduction to the corresponding radical anion. However, this wave is absent in the CV of **2**. Accordingly, treatment of an Et_2_O solution of **1** (green) and **2** (violet) each with two equivalents of GaCl_3_ immediately led to the precipitation of a dark green solid. After workup, the radical cations **3** and **4** were isolated as green crystalline solids (Scheme 1). The use of an excess GaCl_3_ should be avoided as it leads to the over oxidized products, the dications **5** and **6**. Indeed, reactions of **3** and **4** with two equivalents of GaCl_3_ quantitatively gave **5** and **6**, respectively. Alternatively, **5** and **6** are also accessible in one‐pot reaction of **1** or **2** with four equivalents of GaCl_3_.

**Scheme 1:**
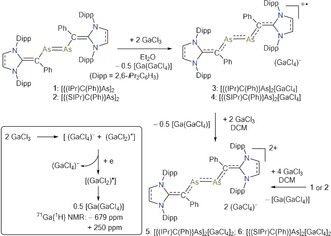
Synthesis of divinyldiarsene radical cations **3** and **4** as well as dications **5** and **6**. Reduction of GaCl_3_ into [Ga(GaCl_4_)] (inset) via disproportionation of the putative GaCl_2_ intermediate.

Two molecules of GaCl_3_ are required for one‐electron oxidation of **1** and **2**. The putative oxidizing species is (GaCl_2_)^+^ that is formed according to 2 GaCl_3_⇄(GaCl_4_)^−^+(GaCl_2_)^+^. The reduction of (GaCl_2_)^+^ yields GaCl_2_, which eventually disproportionates to form the stable mixed‐valence Ga^I^/Ga^III^ compound [Ga(GaCl_4_)] (Supporting Information).^9^ Compounds **3**–**6** are stable under an inert gas atmosphere but readily decompose when exposed to air. Compound **3** and **4** were NMR‐silent, thus indicating their paramagnetic nature. The dications **5** and **6** are red crystalline solids and exhibit well‐resolved ^1^H and ^13^C{^1^H} NMR signals for the N‐heterocyclic vinyl (NHV) moieties (Supporting Information).

Solid‐state molecular structures of **3** (Figure 2), **4** (Figure 3), **5** (Figure 4), and **6** (Supporting Information, Figure F8) were determined by X‐ray diffraction, which exhibit the intact As−As bond with *trans*‐bent geometries along the two‐coordinated arsenic atoms. The HOMO of diarsenes **1** and **2** is the π‐orbital of the As=As bond.^8^ Thus, the formation of **3** and **4** as well as **5** and **6** is the result of sequential one electron removal from the HOMO of **1** and **2**. Clearly, this leads to a steady increase in the As−As bond length of **3** (2.322(1) Å) and **5** (2.419(1) Å) as well as **4** (2.330(1) Å) and **6** (2.414(1) Å) with respect to those of **1** (2.296(1) Å) and **2** (2.290(1) Å) (Table 1). The C2−As1/C4−As2 bond lengths of **3** (1.867(4) Å) and **4** (av. 1.876(2) Å) are shorter compared to that of **1** (1.919(1) Å) and **2** (1.936(3) Å respectively. The C1−C2/C3−C4 bond lengths of **3** (1.424(5) Å) and **4** (av. 1.420(2) Å) are however rather stretched with respect to those of **1** (1.376(2) Å) and **2** (1.369(3) Å). This can be rationalized as the increase of the formal positive charge on the arsenic atoms of **3** and **4** leads to the π‐electron density transfer from the vinylic C=C bond to the arsenic atom.

**Figure 2:**
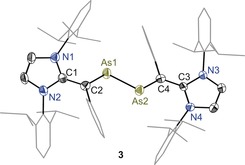
Molecular structure of divinyldiarsene radical cation **3** determined at 100 K. Ellipsoids are set at 50 % probability. Hydrogen atoms, solvent molecules, and the counterion (GaCl_4_) are omitted for clarity.^12^

**Figure 3:**
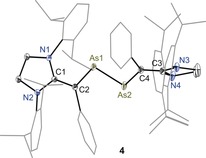
Molecular structure of divinyldiarsene radical cation **4** determined at 100 K. Ellipsoids are set at 50 % probability. Hydrogen atoms, solvent molecules, minor occupied disordered atoms, and the counterion (GaCl_4_) are omitted for clarity.^12^

**Figure 4:**
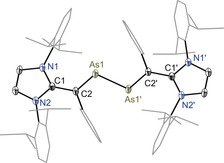
Molecular structure of divinyldiarsene dication **5** determined at 100 K. Ellipsoids are set at 50 % probability. Hydrogen atoms, solvent molecules, and the counterions (GaCl_4_) are omitted for clarity.^12^

**Table 1:** Selected bond lengths [Å] and angles [°] of diarsenes (**1**, **2**) and the corresponding radical cations (**3**, **4**) and dications (**5**, **6**).

	As−As^[a]^	C2−As1 C4−As2	C1−C2 C3−C4	C1−N1/N2 C3−N3/N4	N1‐C1‐N2 N3‐C3‐N4
**1** ^[b]^	2.296(1)	1.919(1)	1.376(2)	1.405(2)/1.395(2)	104.4(2)
**3**	2.322(1)	1.867(4) 1.867(3)	1.424(5) 1.424(4)	1.368(4)/1.364(5) 1.359(4)/1.368(4)	105.7(3) 105.9(3)
**5** ^[b]^	2.419(1)	1.833(3)	1.451(4)	1.354(4)/1.355(4)	107.2(2)
**2** ^[b]^	2.290(1)	1.936(3)	1.369(3)	1.398(3)/1.397(3)	107.3(2)
**4**	2.330(1)	1.873(2) 1.880(2)	1.427(2) 1.412(2)	1.356(2)/1.364(2) 1.370(2)/1.369(2)	109.67(1) 108.94(1)
**6**	2.414(1)	1.839(3) 1.822(3)	1.463(4) 1.469(3)	1.335(3)/1.335(4) 1.334(3)/1.334(3)	111.4(2) 111.5(2)

[a] As1‐As1′/ As1‐As2. [b] Molecular structure features crystallographic center of inversion (*Ci*).

As expected, a more pronounced trend in the elongation of As1−As1′/As2 and C1−C2/C3−C4 bonds while the contraction of the C2−As1/C4−As2 bond lengths of dications **5** and **6** is observed compared to radical cations **3** and **4**. The As−As bond length of **5** (2.414(4) Å) and **6** (2.414(4) Å) is longer than that of radical cations **3** (2.322(5) Å) and **4** (2.330(1) Å), however it is still shorter compared to an As−As single bond (ca. 2.46 Å).^10^ Moreover, the C2−As1/C4−As2 bond lengths of **5** (1.836(3) Å) and **6** (av. 1.830(3) Å) are longer than the C=As double bond length of arsalkenes (1.75–1.79 Å).^11^ The As‐As‐C angle in **1** (99.0(1)°) and **2** (98.7(1)°) is comparable with that of the corresponding radical cations **3** (av. 99.7(1)°) and **4** (98.0(5)°). The same in dications **5** (95.7(9)°) and **6** (av. 96.3(8)°) is however marginally smaller. These features suggest the presence of a conjugated C_2_As_2_C_2_ π‐electron system.

Further insights into the electronic structures of **3**–**6** were obtained by DFT calculations. The optimized geometries of **3**–**6** at the M06‐2X/def2‐TZVPP//M06‐2X/def2‐SVP level of theory (Supporting Information) show good agreement with their solid‐state structures. The computed NPA atomic partial charges (Supporting Information, Table T4) indicate that the As_2_ fragment in **3** (+0.40*e*), **4** (+0.47*e*), **5** (+0.57*e*), and **6** (0.62*e*) carries a positive charge, which is higher than that in **1** (+0.27*e*) and **2** (+0.32*e*).^8^ Each of the vinyl (C2/C4) carbon atoms of **3** (−0.54*e*), **4** (−0.56*e*), **5** (−0.47*e*), and **6** (−0.50*e*) bears a negative, whereas the carbenic carbon (C1/C3) of **3** (+0.42*e*), **4** (+0.52*e*), **5** (+0.41*e*), and **6** (+0.55*e*) bears a positive charge. The WBIs (Wiberg bond indices) for the As−As bond of **3** (1.25), **4** (1.24), **5** (1.00), and **6** (0.99) as well as for the C2/C4−As bonds of **3** (1.21), **4** (1.19), **5** (1.54), and **6** (1.53) indicate the delocalization of π electrons over the C_2_As_2_C_2_ framework. The SOMO (singly occupied molecular orbital) of **3** (Figure 5) and **4** (Supporting Information, Figure F14) is the π‐orbital of the As=As bond, whereas the LUMO (lowest unoccupied molecular orbital) is the π* orbital of the As=As bond. In contrast, the HOMO of **5** (Supporting Information, Figure F15) and **6** (Supporting Information, Figure F16) is the π‐type orbital mainly located at the C_(Ph)_−As bond. The LUMO of **5** and **6** is the π* orbital located at the C_2_As_2_C_2_ unit. The UV/Vis spectrum of **3** (Supporting Information, Figure F2) and **4** (Supporting Information, Figure F3) each exhibits three main absorption bands, which are red‐shifted in comparison to those of **1** and **2**.^8^ Based on TD‐DFT calculations, the band at 822 nm (**3**) and 811 nm (**4**) may be assigned to the SOMO related (S→L and S‐1→L) transitions.

**Figure 5:**
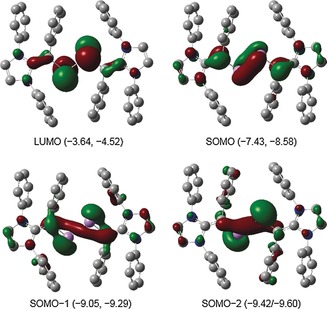
Molecular orbitals (isovalue 0.04) of the radical cation **3** calculated at M06‐2X/def2‐TZVPP//def2‐SVP level of theory with energies (eV) for both (α, β) spin states. Hydrogen atoms as well as isopropyl groups are omitted for clarity.

The EPR spectra of **3** and **4** were recorded in THF at 9.63 GHz. At 298 K, **3** and **4** exhibit a featureless singlet (Supporting Information, Figures F6 and F7) while at 80 K a broadened septet with poorly resolved hyperfine components was observed because of coupling with two magnetically equivalent ^75^As nuclei (Figure 6). These features are similar to those of the radical cation **I** (Figure 1) reported earlier by Robinson and co‐workers.3a The EPR spectra were simulated by using the g values, the hyperfine couplings of each As and *ortho* hydrogen atoms of the phenyl groups, and three linewidth parameters to take into account unresolved hyperfine couplings (Supporting Information, Table T11).

**Figure 6:**
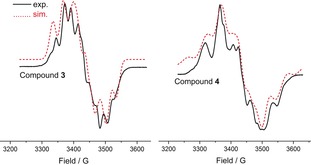
X‐Band EPR spectra of **3** and **4** at 80 K in THF (*ν*=9.63 GHz, Mod. Amp. 5G, *P*_mw_=2 mW).

The calculated Mulliken atomic spin density for **3** and **4** (Figure 7) reveals that the unpaired electron is mainly located at the π‐conjugated CAs_2_C framework (Supporting Information, Table T10). In **3**, 12 % of spin‐density is located at each of the arsenic atoms whereas the spin density at each of the vinylic carbon atoms is 20 %. The spin density at each of the ring nitrogen atoms of **3** and **4** is 5 %. Remarkably, the spin density at the original carbene carbon atom is negligible. In comparison with **3**, the spin density at the arsenic atoms (16 % each) of **4** is higher compared to that of **3** (12 % each). Furthermore, the spin density at the vinylic carbon atoms (15 % each) of **4** is lower with respect to that of **3** (20 % each). This is most likely due to the puckered (non‐planar) structure of 1,3‐imidazoline rings of **4** that twist the vinylic C=C bond out of the As=As bond plane, leading to a diminished π conjugation compared to that in **3** featuring planar 1,3‐imidazole rings. This is also revealed in the X‐ray structures of **3** and **4** (Figures 2 and 3). The C_3_N_2_ ring plane angle of **3** (6.53(14)°) is considerable smaller compared with that of **4** (83.33(11)°). Similarly, the C2‐As1‐As2‐C4 torsion angle of 178.57(14)° in **3** is larger than that in **4** (163.37(8)°).

**Figure 7:**
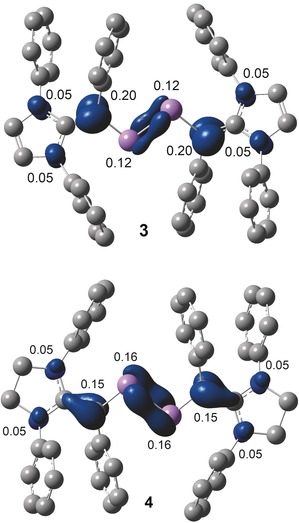
Mulliken spin densities calculated at M06‐2X/def2‐TZVPP//def2‐SVP (isovalue 0.04) of **3** and **4**.

In conclusion, the first diarsene radical cations **3** and **4** as well as the dications **5** and **6** have been prepared as crystalline solids. All compounds **3**–**6** have been characterized by EPR/ NMR and UV/vis spectroscopy, and X‐ray diffraction and analyzed by computational studies. In accessing **3**–**6** from **1** and **2**, GaCl_3_ functions as an oxidizing agent and two equivalents of GaCl_3_ are required for one‐electron oxidation. The formation of mixed‐valence Ga^I^/Ga^III^ compound [Ga(GaCl_4_)] as the main‐side product has been shown with ^71^Ga{^1^H} NMR spectroscopy. Experimental and theoretical results suggest that the radical cations **3** and **4** are stabilized by the delocalization of unpaired electron over the CAs_2_C‐unit. DFT calculations reveal that the spin density is mainly located at the arsenic (12 % in **3** and 16 % in **4** on each As) and vinylic carbon (20 % in **3** and 15 % in **4** on each C) atoms.

## Conflict of interest

The authors declare no conflict of interest.

## Supporting information

As a service to our authors and readers, this journal provides supporting information supplied by the authors. Such materials are peer reviewed and may be re‐organized for online delivery, but are not copy‐edited or typeset. Technical support issues arising from supporting information (other than missing files) should be addressed to the authors.

Supplementary
