# Supplementary material for: Crystalline Divinyldiarsene Radical Cations and Dications
Source: Angew Chem Int Ed Engl. 2019 Oct 23;58(49):17599–603. doi: 10.1002/anie.201909144 (PMC6899687; doi:10.1002/anie.201909144)
Supplement: Supplementary file 1 — Supplementary [file ANIE-58-17599-s001.pdf]

Angewandte Chemie
Eine Zeitschrift der Gesellschaft Deutscher Chemiker

Supporting Information

Crystalline Divinyldiarsene Radical Cations and Dications

*Mahendra K. Sharma, Sebastian Blomeyer, Beate Neumann, Hans-Georg Stammler, Maurice van Gastel, Alexander Hinz, and Rajendra S. Ghadwal**

anie_201909144_sm_miscellaneous_information.pdf

Table of contents

Experimental Section	pp 2-4
Cyclic Voltammetry	pp. 4-6
Copies of NMR Spectra	pp 7-9
UV-Visible Spectroscopy	pp 10-11
EPR Spectroscopy	pp 12
Crystallographic Details	pp 13-16
Computational Details	pp 16-37
References	pp 38

Experimental Section

All the experiments and manipulations were carried out under an inert gas (Ar or N_2) atmosphere using standard *Schlenk* techniques or an MBraun LABmaster Pro glovebox. THF, toluene, and *n*-hexane were dried by refluxing over NaK, distilled prior to use, and stored over molecular 3Å sieve. Starting materials, $[\{(IPr)CPh\}As]_2$ (**1**) and $[\{(SIPr)CPh\}As]_2$ (**2**) were synthesized according to the reported method.[1] $GaCl_3$ was purchased from Sigma Aldrich and used without further purification. NMR spectra for **5** and **6** were recorded using a Bruker Avance III 500HD NMR spectrometer. Chemical shifts are given in δ ppm and are referenced to the solvent residual peaks.[2] UV-visible spectra were recorded using a Thermo Fisher Evolution 300 spectrophotometer. Melting points were measured using a Büchi B-545 melting point apparatus.

Synthesis of $[\{(IPr)C(Ph)\}As]_2(GaCl_4)$ (3). To a *Schlenk* flask containing 50 mL diethyl ether solution of $[\{(IPr)C(Ph)\}As]_2$ (**1**) (300 mg, 0.27 mmol) was added $GaCl_3$ (90 mg, 0.51 mmol) in one portion at room temperature. The resulting suspension was stirring overnight. The precipitate was collected by filtration, washed with 10 mL diethyl ether, and dried in *vaccuo* to afford **3** as a dark green solid. Suitable single crystals for X-ray diffraction studies were grown by a slow diffusion of *n*-hexane into a saturated dichloromethane solution of **3** at room temperature. Yield (210 mg, 59%). Mp: 187 °C (dec.). Elemental analysis (%), calcd for **3**, $C_{68}H_{82}As_2Cl_4GaN_4$ (1316.78): C, 62.02; H, 6.28; N, 4.25; found: C, 62.42; H, 6.61; N, 4.39. UV/Vis (THF, λ (nm) (ε (M^{-1} cm^{-1})): 283 (7690), 308 (7384), 378 (4775), 500 (9287), 822 (1875).

Synthesis of $[\{(SIPr)C(Ph)\}As]_2(GaCl_4)$ (4). To a *Schlenk* flask containing 10 mL diethyl ether solution of $[\{(SIPr)C(Ph)\}As]_2$ (**2**) (215 mg, 0.19 mmol) was added $GaCl_3$ (68 mg, 0.77 mmol) in one portion at room temperature. The resulting suspension was stirred for 10 minutes. The precipitate formed was collected by filtration, washed with 5 mL diethyl ether, and dried in *vaccuo* to afford **4** as a green solid. Suitable single crystals for X-ray diffraction studies were grown by a slow diffusion of *n*-hexane into a saturated dichloromethane solution of **4** at room temperature. Yield (178 mg, 69%). Mp: 208 °C (dec.). Elemental analysis (%), calcd for **4**, $C_{68}H_{86}As_2Cl_4GaN_4$ (1320.82): C, 61.84; H, 6.56; N, 4.24; found: C, 62.09; H, 6.71; N, 4.41. UV/Vis (THF, λ (nm) (ε (M^{-1} cm^{-1})): 291 (8713), 372 (7618), 801 (1801).

Synthesis of [{(IPr)C(Ph)}As]$_2$(GaCl$_4$)$_2$ (5) from 3. To a 20 mL dichloromethane solution of **3** (150 mg, 0.11 mmol) was added $GaCl_3$ (40 mg, 0.23 mmol) at room temperature. The green colour of solution turned immediately dark red. The resulting solution was stirred for 6h. The volatiles were removed under reduced pressure to get compound **5** as a dark red solid. Single crystals suitable for X-ray diffraction studies were grown by a slow diffusion of *n*-hexane into a saturated THF solution of **5**. Yield: 170 mg, 98%. Mp: 244 °C (dec.). Elemental analysis (%), calcd for **5**, $C_{68}H_{82}As_2Cl_8Ga_2N_4$ (1528.32): C, 53.44; H, 5.41; N, 3.67; found: C 53.26; H 5.31; N 3.49. ^{1}H NMR (CD_2Cl_2, 298 K, 500 MHz): δ = 7.69 (s, 4H, NC*H*), 7.45 (t, *J* = 7.8 Hz, 4H, C_6H_3), 7.18 (t, *J* = 7.5 Hz, 2H, C_6H_3), 7.13 (d, *J* = 7.8 Hz, 8H, C_6H_3, C_6H_5), 7.07 (t, *J* = 7.6 Hz, 4H, C_6H_5), 6.33 (d, *J* = 7.4 Hz, 4H, C_6H_5), 2.25-2.19 (m, 8H, C*H*(CH_3)$_2$), 1.13 (d, *J* = 6.7 Hz, 24H, CH(CH_3)$_2$), 0.94 (d, *J* = 6.7 Hz, 24H, CH(CH_3)$_2$). ^{13}C{^{1}H} NMR (CD_3CN, 298 K, 125 MHz): δ = 147.4, 146.3, 133.4 (*C*CAs, *ipso*-C_6H_3;*o*-C_6H_3, *ipso*-C_6H_5); 130.9, 130.6, 130.1, 129.9, 129.2, 128.2, 127.6, 126.2, 125.8 (*m*-, *p*-C_6H_3, *o*-, *m*-, *p*-C_6H_5); 30.0, 25.9, 22.8 (*C*H(*C*H$_3$)$_2$). UV/Vis (THF, λ (nm) (ε (M^{-1} cm^{-1})): 291 (2691), 309 (2610), 417 (2718), 490 (3005).

One-pot Synthesis of [{(IPr)C(Ph)}As]$_2$(GaCl$_4$)$_2$ (5). Compound **5** was synthesized following the similar protocol used for compound **3**, using **1** (300 mg, 0.27 mmol), $GaCl_3$ (191 mg, 1.08 mmol), and 50 mL toluene. Yield (311 mg, 76%).

Synthesis of [{(SIPr)C(Ph)}As]$_2$(GaCl$_4$)$_2$ (6) from 4. To a 20 mL dichloromethane of **4** (200 mg, 0.15 mmol) was added $GaCl_3$ (54 mg, 0.31 mmol) at room temperature. The green colour of solution turned immediately red, which was stirred for 6h. The volatiles were removed under reduced pressure to get compound **6** as a red solid. Single crystals suitable for X-ray diffraction studies were grown by a slow diffusion of *n*-hexane into a saturated THF solution of **6**. Yield: 230 mg, 99%. Mp: 241 °C (dec.). Elemental analysis (%), calcd for **6**, $C_{68}H_{86}As_2Cl_8Ga_2N_4$ (1532.35): C, 53.30; H, 5.66; N, 3.66; found: C 53.09; H 5.49; N 3.81. ^{1}H NMR (CD_2Cl_2, 298 K, 500 MHz): δ = 7.34 (t, *J* = 8.2 Hz, 4H, *p*-C_6H_3), 7.18 (t, 2H, *J* = 7.2 Hz, *m*-C_6H_3), 7.05 (m, 12H, C_6H_3, C_6H_5), 6.32 (d, *J* = 7.6 Hz, 4H, C_6H_5), 4.39 (s, 8H, CH_2), 2.74-2.79 (m, 8H, C*H*(CH_3)$_2$), 1.23 (d, *J* = 6.7 Hz, 24H, CH(CH_3)$_2$), 0.88 (d, *J* = 6.6 Hz, 24H, CH(CH_3)$_2$). ^{13}C{^{1}H} NMR (CD_3CN, 298 K, 125 MHz): δ = 192.2, 168.5, 147.4,146.1, 132.6, 131.6, 131.0, 130.5, 130.0, 127.7, 126.2 (*CC*As, *C*$_6$H$_3$, *C*$_6$H$_5$), 54.2 (*C*H$_2$), 29.7, 26.7, 23.7 (CH(*C*H$_3$)$_2$). UV/Vis (toluene, λ (nm) (ε (M^{-1} cm^{-1})): 290 (12054), 358 (9169).

One-pot Synthesis of [{(SIPr)C(Ph)}As]$_2$(GaCl$_4$)$_2$ (6). Compound **6** was synthesized following the similar protocol used for compound **3**, using **2** (286 mg, 0.26 mmol), $GaCl_3$ (182 mg, 1.03 mmol, and 40 mL toluene. Yield (310 mg, 78.4%).

Cyclic Voltammetry

Cyclic voltammetry (CV) experiments were carried out using a PGSTAT 101 electrochemical workstation (METROHM). All experiments were carried out inside a glovebox under argon atmosphere. The setup consisted of a glassy carbon working electrode (surface area = 0.04 cm^2), a glassy carbon counter electrode, and a platinum wire pseudo-reference electrode. The recorded voltammograms were referenced to the internal standard Fc^+/Fc (ferrocenium/ferrocene) couple.

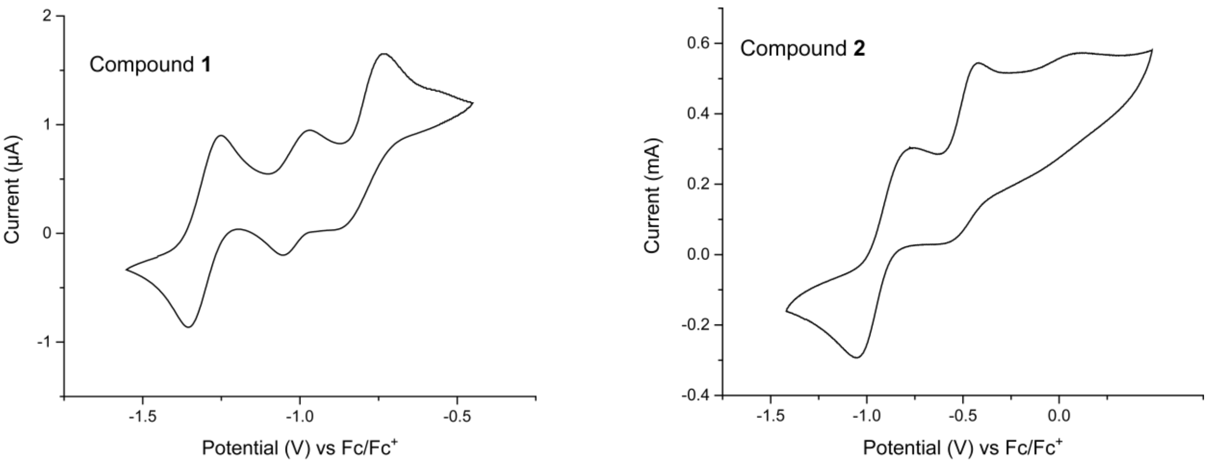

Figure F1a. Cyclic voltammograms (CVs) of compounds **1** and **2** in dichloromethane (in 0.01 M *n*-$Bu_4N[Al(OC(CF_3)_3]_4$, 100 mVs^{-1}, vs Fc/Fc^+).

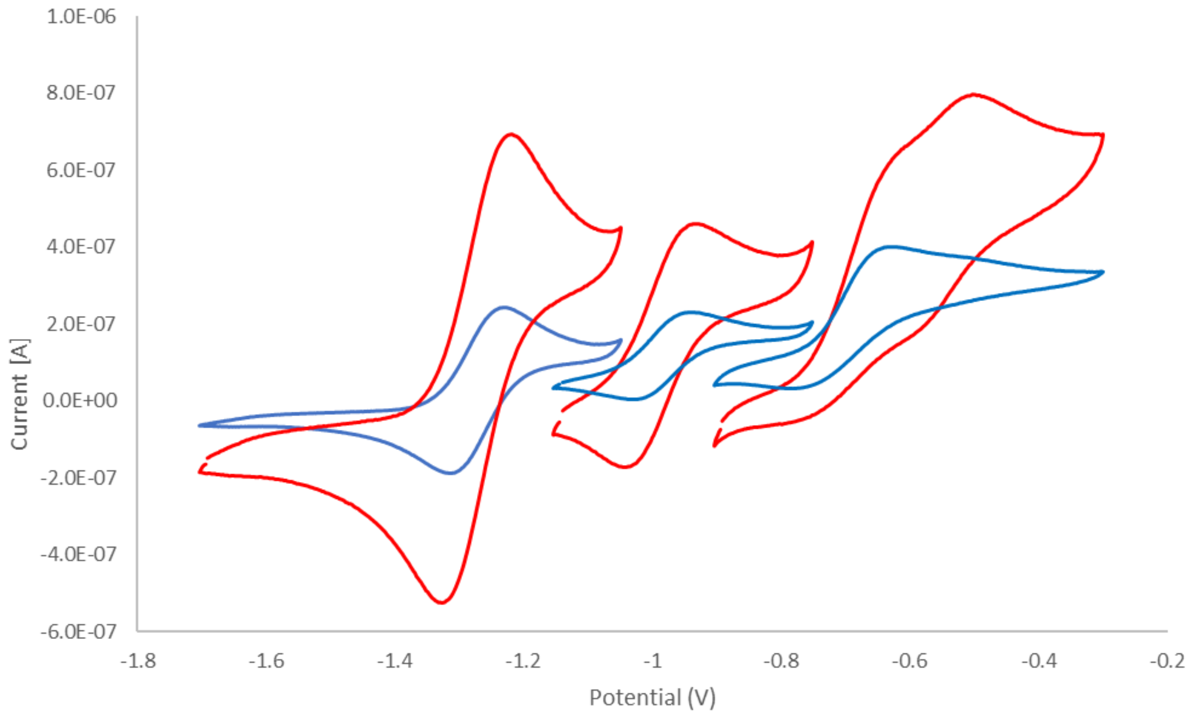

Figure F1b. Cyclic voltammograms (CVs) of compound **1** in dichloromethane (in 0.01 M *n*-$Bu_4N[Al(OC(CF_3)_3]_4$, 50 (blue) and 500 mVs^{-1} (red), scan towards positive potential first, vs Fc/Fc^+).

Redox events:

–1.28 V – quasi-reversible ($\Delta E_{Imax/Imin,50mV/s}$: 81 mV, $\Delta E_{Imax/Imin,500mV/s}$: 105 mV)

–1.00 V – quasi-reversible ($\Delta E_{Imax/Imin,50mV/s}$: 85 mV, $\Delta E_{Imax/Imin,500mV/s}$: 110 mV)

–0.67 V – nearly irreversible ($\Delta E_{Imax/Imin,50mV/s}$: 159 mV, $\Delta E_{Imax/Imin,500mV/s}$: n.d.); at 500 mV/s the reduction half wave disappeared; rapid changes of the redox wave upon repeated scanning were observed with concomitant deposition of presumably elemental As on the electrode.

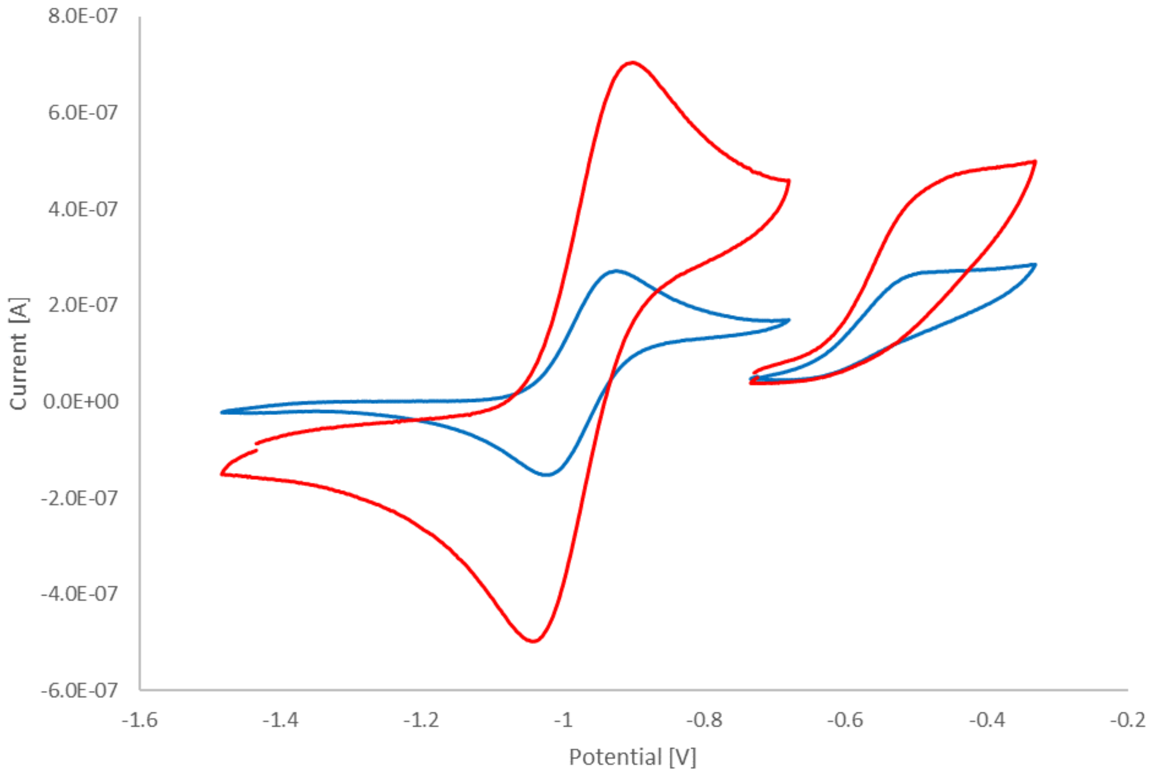

Figure F1c. Cyclic voltammograms (CVs) of compound **2** in dichloromethane (in 0.01 M *n*-$Bu_4N[Al(OC(CF_3)_3]_4$, 50 (blue) and 500 (red) mVs^{-1}, scan towards positive potential first, vs Fc/Fc^+).

Redox events:

–0.92 V – quasi-reversible ($\Delta E_{Imax/Imin,50mV/s}$: 102 mV, $\Delta E_{Imax/Imin,500mV/s}$: 139 mV)

–0.51 V – nearly irreversible ($\Delta E_{Imax/Imin,50mV/s}$: 205 mV, $\Delta E_{Imax/Imin,500mV/s}$: n.d.) at 500 mV/s the reduction half wave disappeared; rapid changes of the redox wave upon repeated scanning were observed with concomitant deposition of presumably elemental As on the electrode.

Copies of NMR Spectra

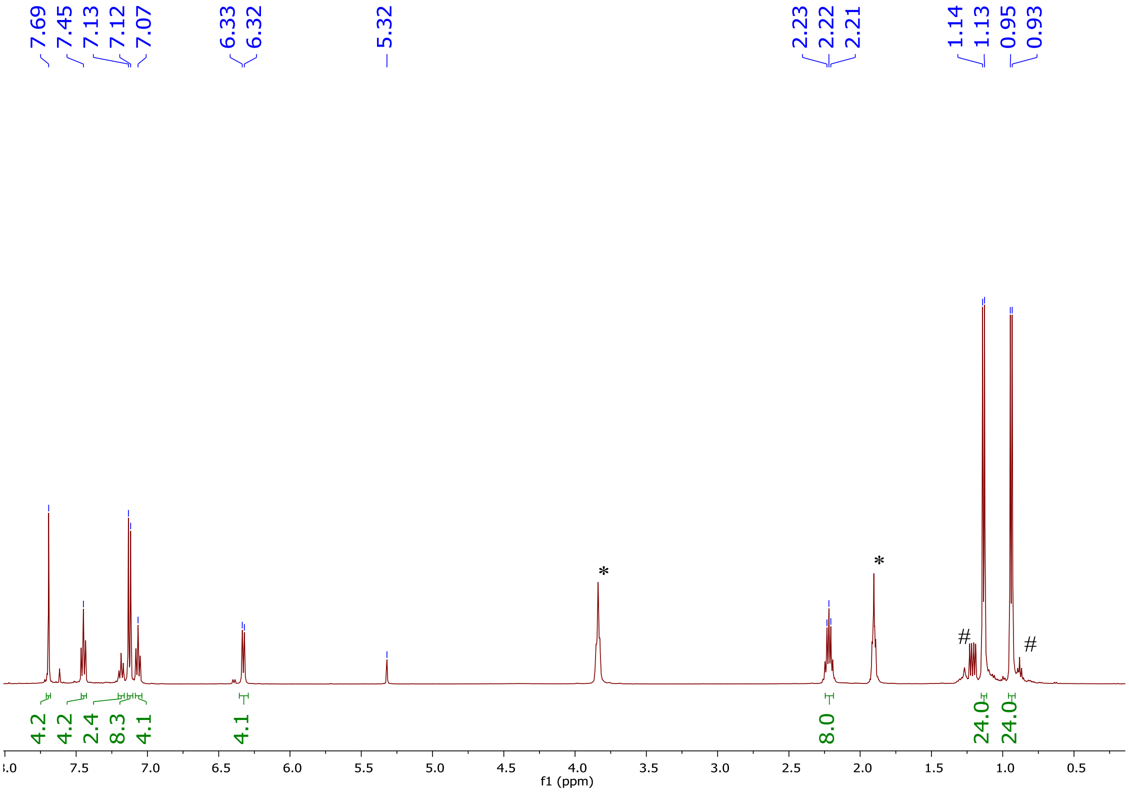

Plot P1. ^{1}H NMR (CD_2Cl_2, 298 K, 500 MHz) spectrum of **5** (*THF, $^{\#}$*n*-hexane).

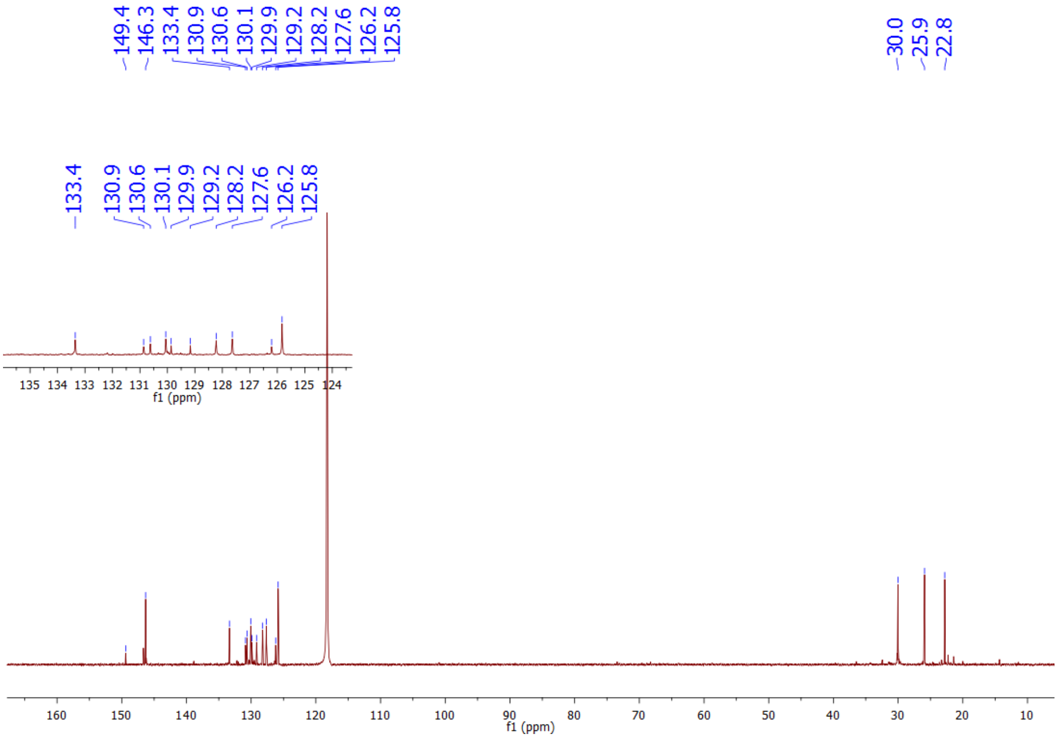

Plot P2. $^{13}C\{^{1}H\}$ NMR (CD_3CN, 298 K, 125 MHz) spectrum of **5**.

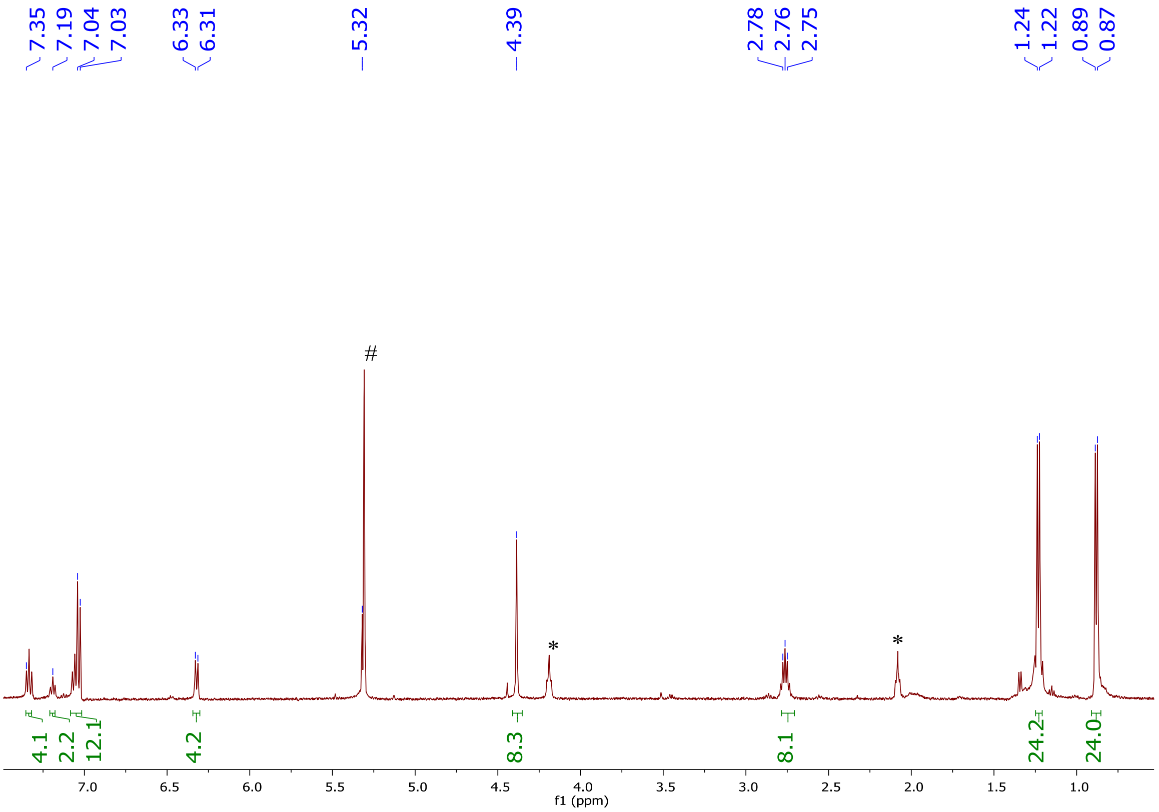

Plot P3. ^{1}H NMR (CD_2Cl_2, 298 K, 500 MHz) spectrum of **6** (*THF, $^{\#}CH_2Cl_2$).

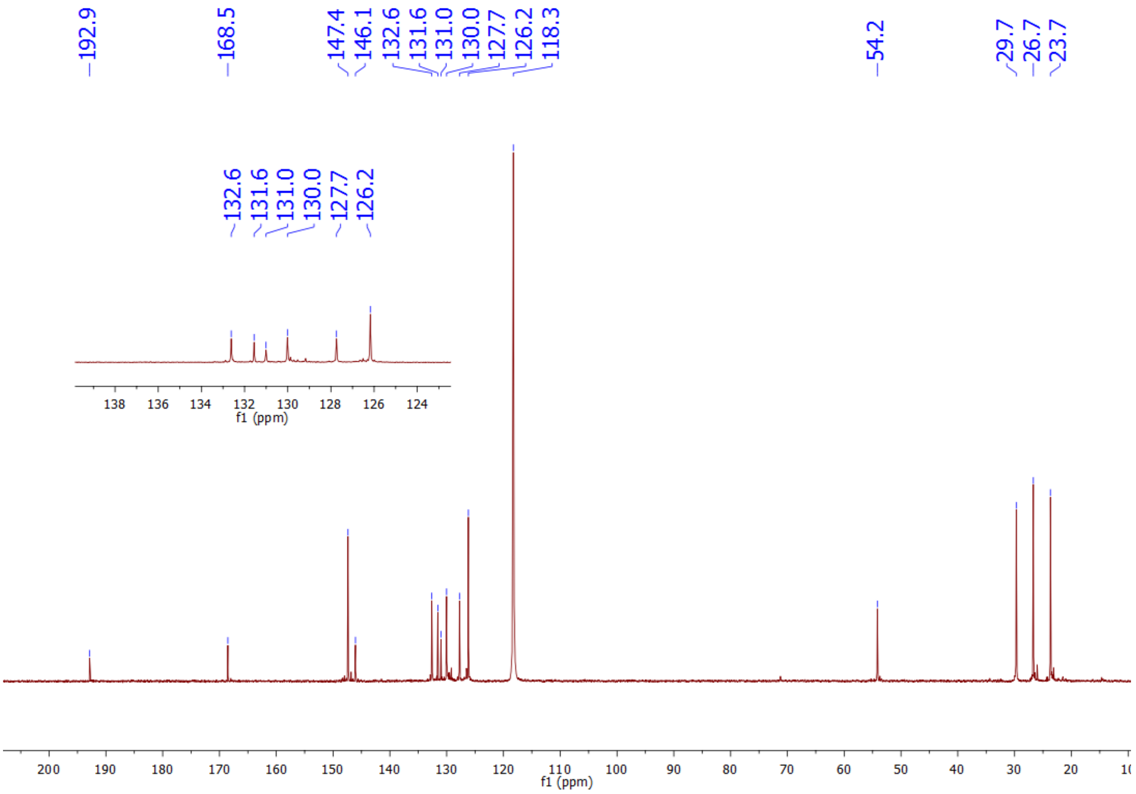

Plot P4. $^{13}C\{^{1}H\}$ NMR (CD_3CN, 298 K, 125 MHz) spectrum of **6**.

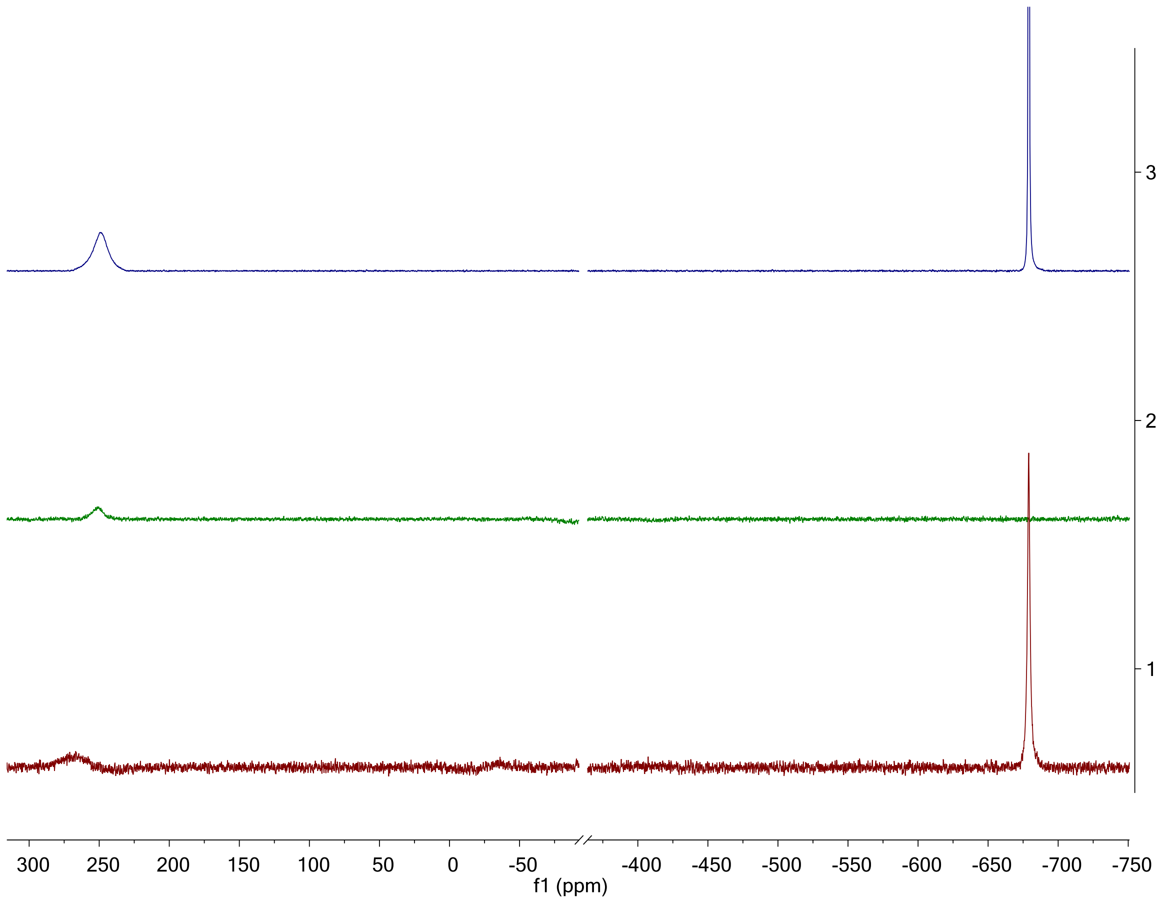

Plot P5. $^{71}Ga\{^1H\}$ NMR spectrum: (1) Toluene soluble part of the reaction of **2** with $GaCl_3$ to give **4** (toluene insoluble) and $[Ga(GaCl_4)]$ (C_6D_6, 298 K, 183 MHz), (2) Dication **6** (CD_2Cl_2, 298 K, 183 MHz), and (3) A freshly prepared $[Ga(GaCl_4)]$ compound (C_6D_6, 298 K, 183 MHz). The signal at ~ –679 ppm corresponds to the $[Ga(I)(C_6D_6)_n]$, whereas the signal at ~ + 250 ppm relates to the $(GaCl_4)^-$ anion (Please see: H. Schmidbaur, *Angew. Chem. Int. Ed.*, 1985, **24**, 893–904 for further detail).

UV-Visible Spectroscopy

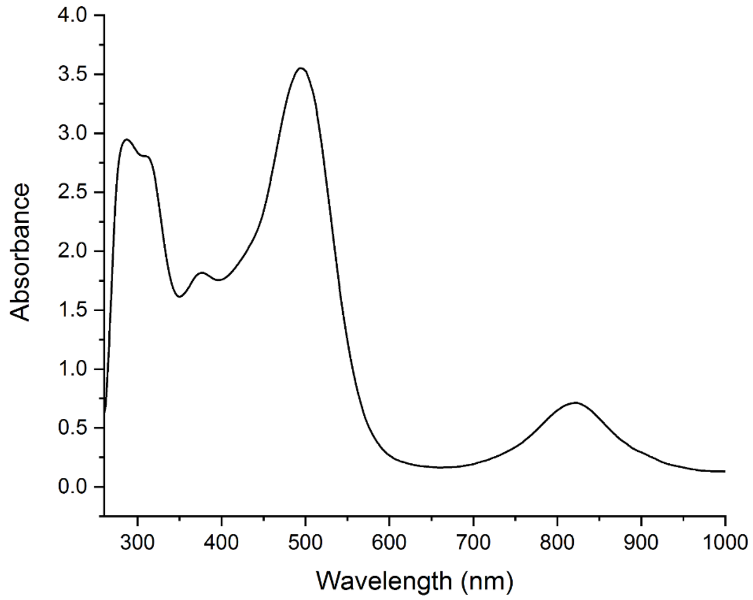

Figure F2. UV-visible spectrum of **3** (3.8 x 10^{-4} M) in THF.

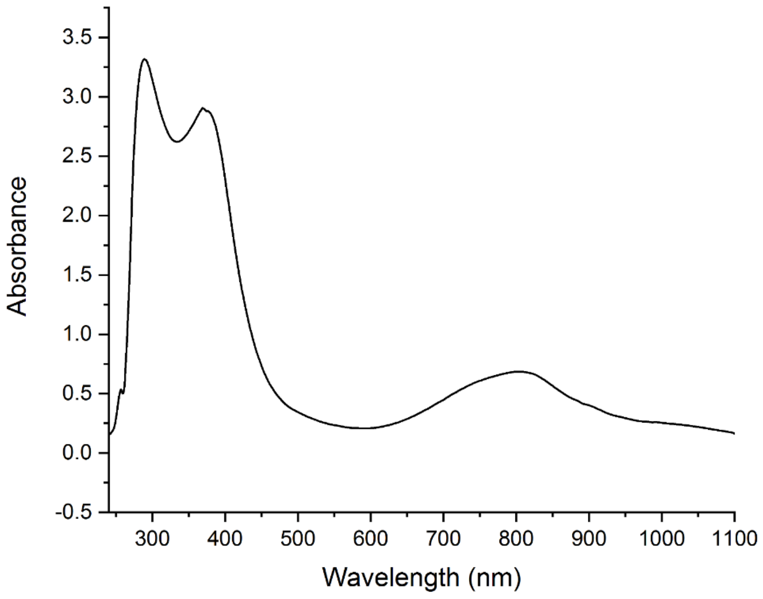

Figure F3. UV-visible spectrum of **4** (3.8 x 10^{-4} M) in THF.

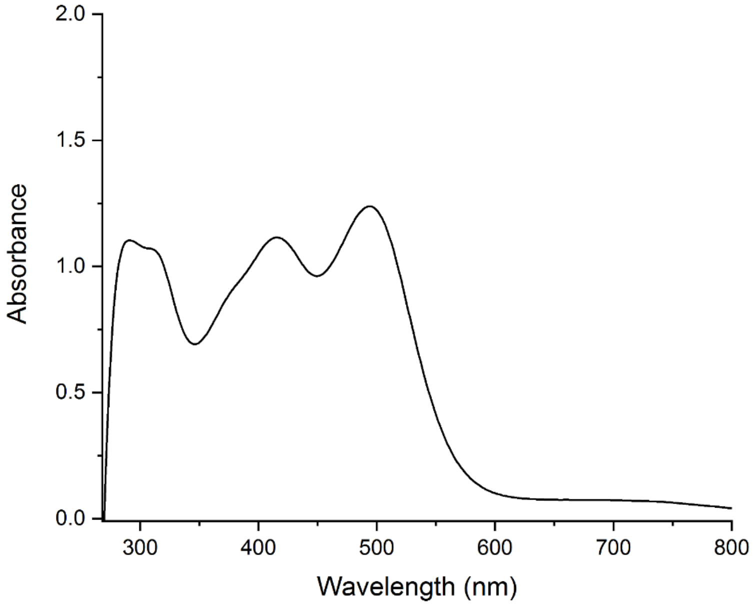

Figure F4. UV-visible spectrum of **5** (4.1 x 10^{-4} M) in THF.

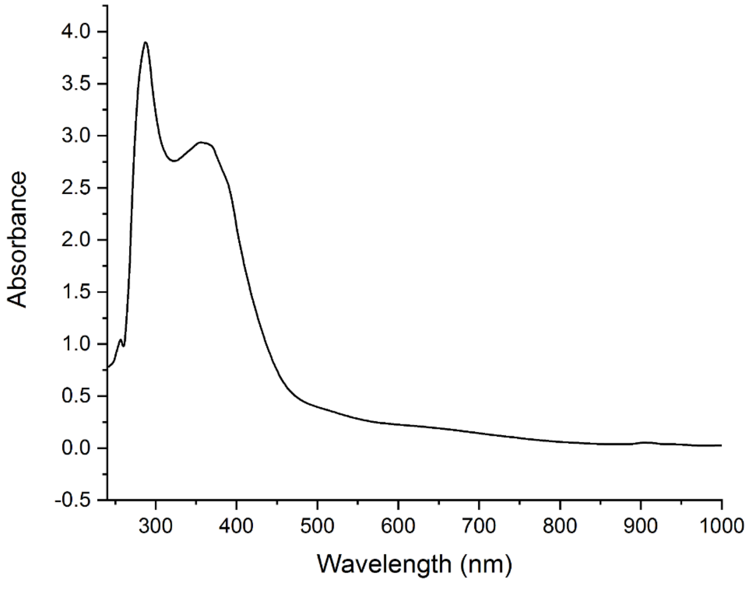

Figure F5. UV-visible spectrum of **6** (3.2 x 10^{-4} M) in THF.

EPR Spectroscopy

The continuous wave (CW) EPR experiments were performed at room temperature (298 K) in a Bruker standard ST9402 resonator and with a Bruker ELEXSY E500 spectrometer. The microwave frequency was 9.63 GHz and the modulation amplitude was 0.3 mT.

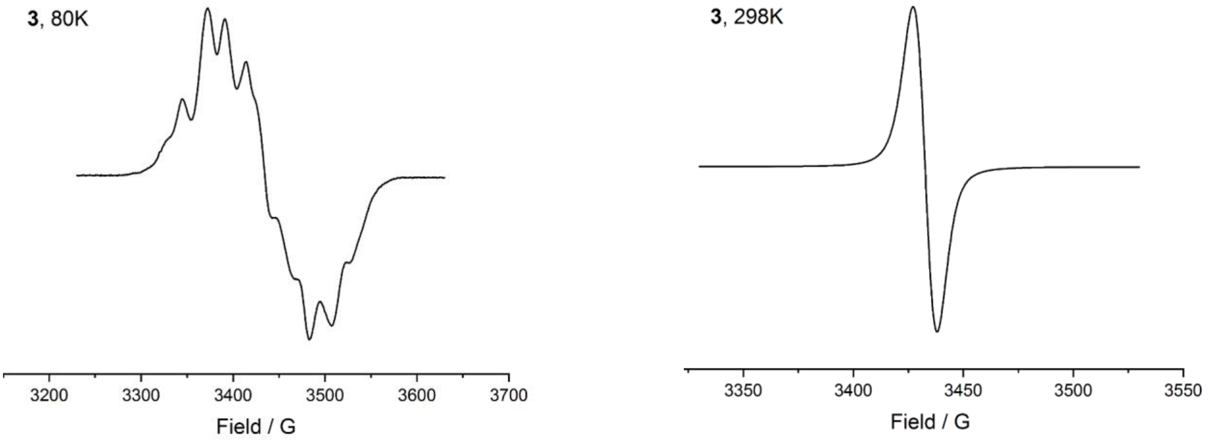

Figure F6. EPR spectra of **3** (4 mM THF solution). Microwave freq. 9.63 GHz, power = 2 mW, mod. freq. 100 KHz.

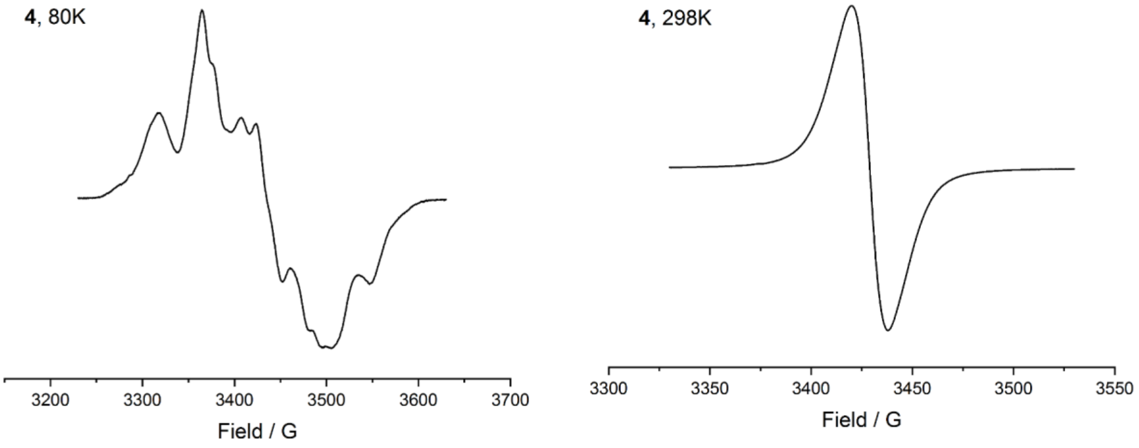

Figure F7. EPR spectra of **4** (4 mM THF solution). Microwave freq. 9.63 GHz, power = 2 mW, mod. freq. 100 KHz.

Crystallographic Details

The single crystal data were examined on a Rigaku Supernova diffractometer using either MoKα (λ = 0.71073 Å) or CuKα (λ = 1.54184 Å) radiation. The crystals were kept at 100.0(1) K during data collection. Using Olex2[3], the structures were solved with the ShelXT[4] structure solution program using Intrinsic Phasing and refined with the ShelXL[5] refinement package using Least Squares minimization. Hydrogen atoms were taken into account using a riding model.

Table T1. Crystal data and structure refinement parameters for compounds **3** and **4**.

	**3** x 2 CH_2Cl_2	**4** x CH_2Cl_2
Empirical formula	$C_{70}H_{86}As_2Cl_8GaN_4$	$C_{70}H_{90}As_2Cl_8GaN_4$
Formula weight	1486.58	1490.61
Temperature/K	100.0(1)	100.0(1)
Crystal system	triclinic	triclinic
Space group	P-1	P-1
a/Å	11.0132(3)	13.2299(3)
b/Å	16.0708(4)	15.5368(3)
c/Å	21.5277(4)	19.3733(3)
α/°	101.1269(18)	78.4248(17)
β/°	91.5147(19)	71.1813(17)
γ/°	97.200(2)	87.8387(16)
Volume/$Å^3$	3704.07(15)	3691.10(13)
Z	2	2
$\rho_{calc}g/cm^3$	1.333	1.341
μ/mm^{-1}	4.467	1.593
F(000)	1534.0	1542.0
Crystal size/mm^3	0.224 × 0.111 × 0.026	0.203 × 0.147 × 0.082
Radiation/Å	Cu Kα (λ = 1.54184)	MoKα (λ = 0.71073)
2θ range for data collection/°	5.654 to 154.192	3.144 to 64.272
Index ranges	$-13 \leq h \leq 13$, $-20 \leq k \leq 20$, $-27 \leq l \leq 27$	$-19 \leq h \leq 19$, $-23 \leq k \leq 23$, $-28 \leq l \leq 28$
Reflections collected	60603	110677
Independent reflections	14865 [R_{int} = 0.0526, R_{sigma} = 0.0369]	24110 [R_{int} = 0.0416, R_{sigma} = 0.0386]
Reflections with $I > 2\sigma(I)$	12324	18837
Data/restraints/parameters	14865/1/807	24110/0/795
Goodness-of-fit on F^2	1.174	1.020
Final R indexes [$I > 2\sigma(I)$]	R_1 = 0.0550, wR_2 = 0.1572	R_1 = 0.0394, wR_2 = 0.0851
Final R indexes [all data]	R_1 = 0.0675, wR_2 = 0.1768	R_1 = 0.0579, wR_2 = 0.0923
Largest diff. peak/hole / e $Å^{-3}$	0.81/-0.98	0.94/-0.99
CCDC number	1939893	1939894

Table T2. Crystal data and structure refinement parameters for compounds **5** and **6**.

	**5** x 6 CH_2Cl_2	**6** x 3 THF
Empirical formula	$C_{74}H_{94}As_2Cl_{20}Ga_2N_4$	$C_{80}H_{110}As_2Cl_8Ga_2N_4O_3$
Formula weight	2037.81	1748.59
Temperature/K	100.0(1)	100.0(1)
Crystal system	monoclinic	triclinic
Space group	$P2_1/n$	P-1
a/Å	14.31710(10)	15.0990(2)
b/Å	15.76120(10)	16.1505(2)
c/Å	20.1159(2)	18.0877(2)
α/°	90	93.7150(10)
β/°	90.9570(10)	92.7440(10)
γ/°	90	104.6160(10)
Volume/Å^3	4538.61(6)	4249.66(9)
Z	2	2
ρ_{calc}g/cm^3	1.491	1.367
μ/mm^{-1}	7.278	4.318
F(000)	2068.0	1812.0
Crystal size/mm^3	0.308 × 0.289 × 0.08	0.371 × 0.124 × 0.039
Radiation/Å	CuKα (λ = 1.54184)	CuKα (λ = 1.54184)
2θ range for data collection/°	7.126 to 153.74	4.906 to 153.572
Index ranges	$-18 \le h \le 18$, $-19 \le k \le 19$, $-25 \le l \le 25$	$-17 \le h \le 18$, $-20 \le k \le 20$, $-22 \le l \le 22$
Reflections collected	161099	156869
Independent reflections	9513 [R_{int} = 0.0655, R_{sigma} = 0.0177]	17753 [R_{int} = 0.0433, R_{sigma} = 0.0207]
Reflections with $I > 2\sigma(I)$	9053	15832
Data/restraints/parameters	19513/2/476	17753/26/950
Goodness-of-fit on F^2	1.096	1.038
Final R indexes [$I > 2\sigma(I)$]	R_1 = 0.0452, wR_2 = 0.1260	R_1 = 0.0481, wR_2 = 0.1303
Final R indexes [all data]	R_1 = 0.0469, wR_2 = 0.1275	R_1 = 0.0533, wR_2 = 0.1356
Largest diff. peak/hole / e Å^{-3}	0.66/-0.97	3.31/-0.96
CCDC number	1939895	1939896

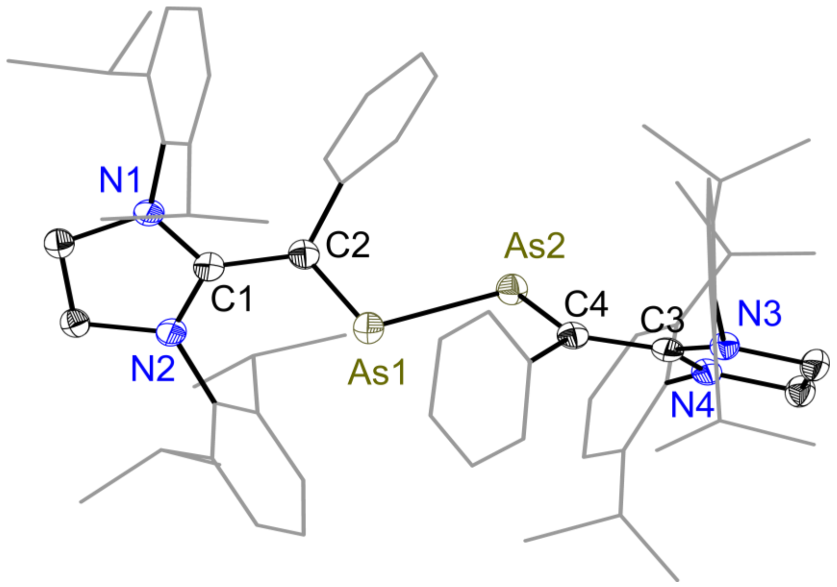

Figure F8. Solid-state molecular structure of dication **6**. Anisotropic displacement parameters are depicted at the 50%probability level. Hydrogen atoms, solvent molecules, and the counter anion $GaCl_4$ have been omitted for clarity. Selected bond lengths (Å) and bond angles (°): As1−As2 2.414(1), As1−C2 1.839(3), As2−C4 1.822(3), C1−C2 1.463(4), C3−C4 1.469(3); C2−As1−As2 97.47(8), C4−As2−As1 95.15(8), C1−C2−As1 117.3(2), C3−C4−As2 119.87(18), N1−C1−N2 111.4(2), N3−C3−N4 111.5(2).

Computational Details

All geometries were optimized with the Gaussian 16 program suite[6] using the DFT functional M06-2X[7] in combination with the Ahlrichs def2-SVP[8] basis function as implemented. The stationary points were located with the Berny algorithm using redundant internal coordinates. Analytical Hessians were computed to determine the nature of stationary points (zero imaginary frequencies for minima).[9] The electronic energies were improved by single point calculations at the M06-2X/def2-TZVPP level of theory.

The Wiberg Bond Indices (WBI)[10] and NPA[11] atomic partial charges have been calculated at the M06-2X/def2-TZVPP//M06-2X/def2-SVP level of theory using the NBO 3.1 interface of Gaussian.[12]

Time-dependent density functional theory (Full-TDDFT) was employed to calculate excitation energies as implemented in ORCA 4.1.1.[13] We used the functional M06-2X in combination the def2-SVP basis sets. The solvent (THF) was described by the conductor-like polarizable continuum model, CPCM.[14]

EPR parameters like *g*-factor and hyperfine coupling constants were calculated at the minimum geometries from M06-2X/def2-SVP calculations using the TPSS functional in combination with both, decontracted and ZORA recontracted def2-TZVP basis sets, as implemented in ORCA 4.1.1.

Table T3. Electronic energies of selected MOs of compounds **3, 4**, **5** and **6** calculated at M06-2X/def2-TZVPP//M06-2X/def2-SVP level of theory. For the radical cations **3** and **4** energies for both spin orbitals are given (α/β).

**orbital**	**energy / eV**			
	**3**	**4**	**5**	**6**
L+1	−2.01/−3.09	−2.06/−3.34	−6.19	−6.32
L	−3.64/−4.52	−4.02/−4.91	−7.42	−7.57
S/H	−7.43/−8.58	−7.91/−9.01	−11.75	−11.93
S/H−1	−9.05/−9.29	−9.28/−9.54	−12.28	−12.25
S/H−2	−9.42/−9.60	−9.66/−9.62	−12.30	−12.26
S/H−3	−9.64/−9.70	−9.77/−9.89	−12.36	−12.27
S/H−4	−9.93/−10.08	−9.97/−9.91	−12.36	−12.32
SOMO/HOMO–LUMO gap	3.79	3.89	4.33	4.36

Table T4. Wiberg bond indices (WBI) and natural population analysis (NPA) atomic charges of compounds **3, 4**, **5** and **6** calculated at M06-2X/def2-TZVPP//def2-SVP level of theory.

**bond**	**WBI**			
	**3**	**4**	**5**	**6**
As–As	1.25	1.24	1.00	0.99
As–C(C–Ph)	1.21	1.19	1.54	1.53
C(C–Ph)–C(IPr/SIPr)$_{ipso}$	1.23	1.27	1.09	1.07
C(IPr/SIPr)$_{ipso}$–N(IPr/SIPr)	1.19	1.18	1.25	1.30
N(IPr/SIPr)$_{ipso}$–C(IPR/SIPr)	1.12	0.94	1.16	0.94
C(IPr/SIPr)–C(IPr/SIPr)	1.66	1.00	1.61	1.00
**atom**	**NPA atomic charge**			
	**3**	**4**	**5**	**6**
As	+0.40	+0.47	+0.57	+0.62
C(C–Ph)	−0.54	−0.56	−0.47	−0.50
C(IPr/SIPr)$_{ipso}$	+0.43	+0.52	+0.41	+0.55
N(IPr/SIPr)	−0.36	−0.43	−0.32	−0.38
C(IPr/SIPr)	−0.06	−0.21	−0.04	−0.22

Table T5. First and second ionization potentials (IP) calculated at M06-2X/def2-TZVPP//M06-2X/def2-SVP level of theory,[15] taking into account the solvation effect with PCM(dichloromethane) and referenced to E^0_{SHE} of −4.28 V.[16] (all energies in a.u., all potentials in V; E_{elec}: electronic energy; E_{solv}: solvation corrected electronic energy; G_{corr}: thermal correction to Gibbs free energy; G_{solv}: solvation corrected Gibbs free energy).

	IPr			SIPr		
	**1**	**3**	**5**	**2**	**4**	**6**
E_{elec}	−7331.24818	−7331.07828	−7330.80607	−7333.66552	−7333.47833	−7333.19394
E_{solv}	−7331.26218	−7331.12170	−7330.95022	−7333.67982	−7333.52278	−7333.33988
G_{corr}	1.23387	1.23124	1.23398	1.28001	1.28001	1.27872
G_{solv}	−7330.02832	−7329.89046	−7329.71624	−7332.39981	−7332.24271	−7.332.06116
Ist IP	−0.53 V (*vs* SHE)			−0.01 (*vs* SHE)		
IInd IP	+0.46 (*vs* SHE)			+0.66 (*vs* SHE)		

Table T6. Wavelength (λ), oscillator strength (f) and main assignment of the TD-PCM(thf)/M06-2X/def2-SVP results for compound **3**; threshold for printing excitations was chosen to be $f \geq 0.04$.

**state no.**	***λ* / nm**	***f***	**Assignment**
**2**	786.3	0.3766	S−1 → S (c = 0.5015)
**3**	567.6	0.0466	S−2 → S (c = 0.9089)
**12**	348.9	0.0464	S−5 → S (c = 0.2401)
**20**	326.7	0.0466	S → L+4 (c = 0.3541)

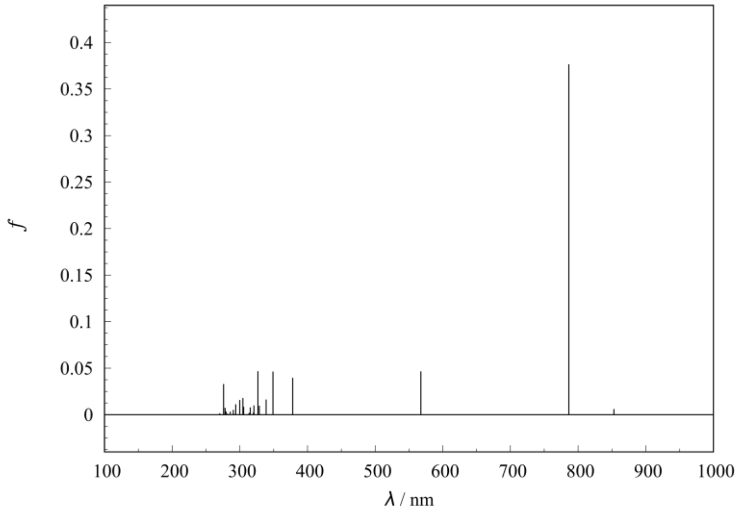

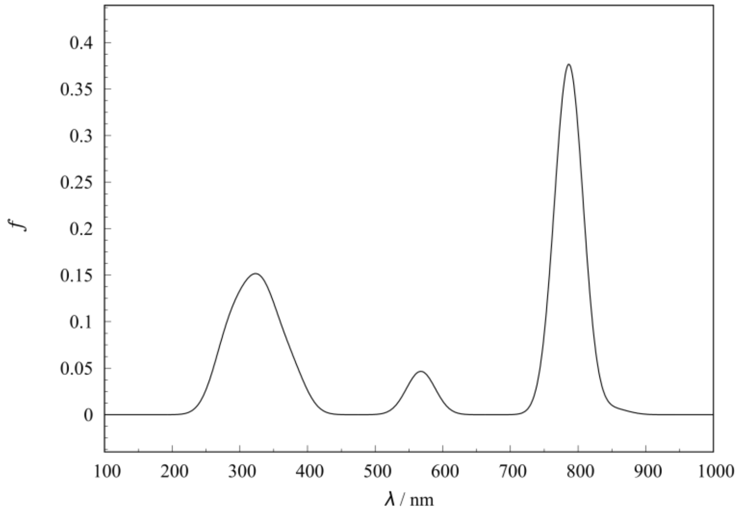

Figure F9. UV-visible spectrum (lines: top; Gaussian line broadening with a full width at half maximum (fwhm) of 50 cm^{-1}: bottom) of **3** calculated at TD-PCM(thf)/M06-2X/def2-SVP.

Table T7. Wavelength (λ), oscillator strength (*f*) and main assignment of the TD-PCM(thf)/M06-2X/def2-SVP results for compound **4**; threshold for printing excitations was chosen to be $f \geq 0.03$.

state no.	λ / nm	*f*	Assignment
1	878.0	0.1342	S−1 → S (*c* = 0.8758)
2	878.7	0.1575	S → L (*c* = 0.9580)
3	815.5	0.0549	S−2 → S (*c* = 0.8534)
15	400.0	0.0371	S → L+1 (*c* = 0.3166)
38	336.7	0.0327	S−4 → L (*c* = 0.1953)

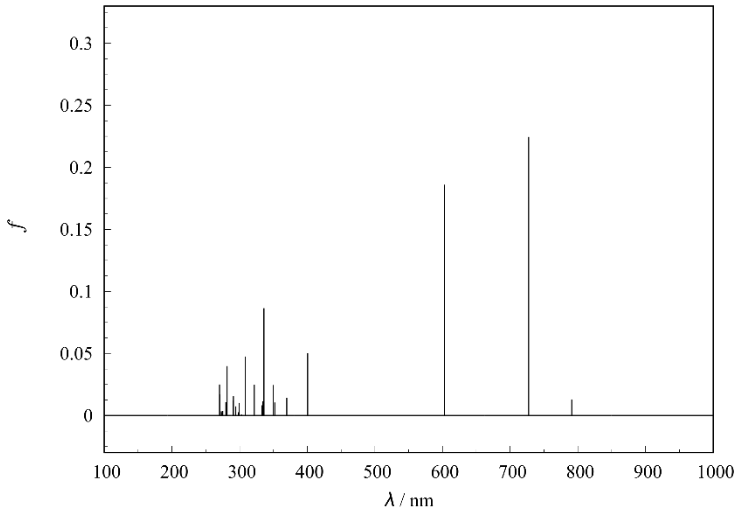

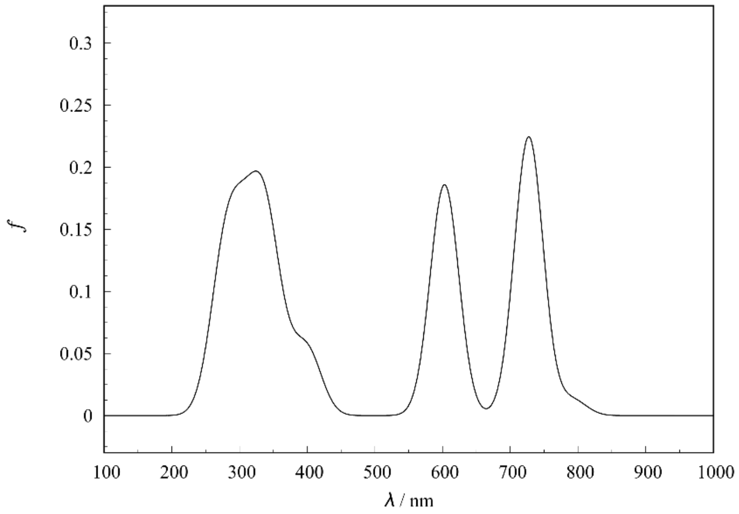

Figure F10. UV-visible spectrum (lines: top; Gaussian line broadening with a fwhm of 50 cm^{-1}: bottom) of **4** calculated at TD-PCM(thf)/M06-2X/def2-SVP.

Table T8. Wavelength (λ), oscillator strength (f) and main assignment of the TD-PCM(thf)/M06-2X/def2-SVP results for compound **5**; threshold for printing excitations was chosen to be $f \geq 0.05$.

**state no.**	λ **/ nm**	f	**Assignment**
1	548.8	0.0716	H → L (c = 0.5076)
2	548.0	0.3088	H → L (c = 0.4609)
7	383.1	0.0804	H−8 → L (c = 0.5056)
19	339.4	0.0876	H−7 → L+1 (c = 0.3064)
31	301.3	0.1032	H → L+4 (c = 0.6573)
32	301.1	0.0524	H−1 → L+2 (c = 0.1367)
37	275.1	0.0815	H−11 → L+1 (c = 0.2261)
47	252.9	0.1254	H−1 → L+2 (c = 0.4021)

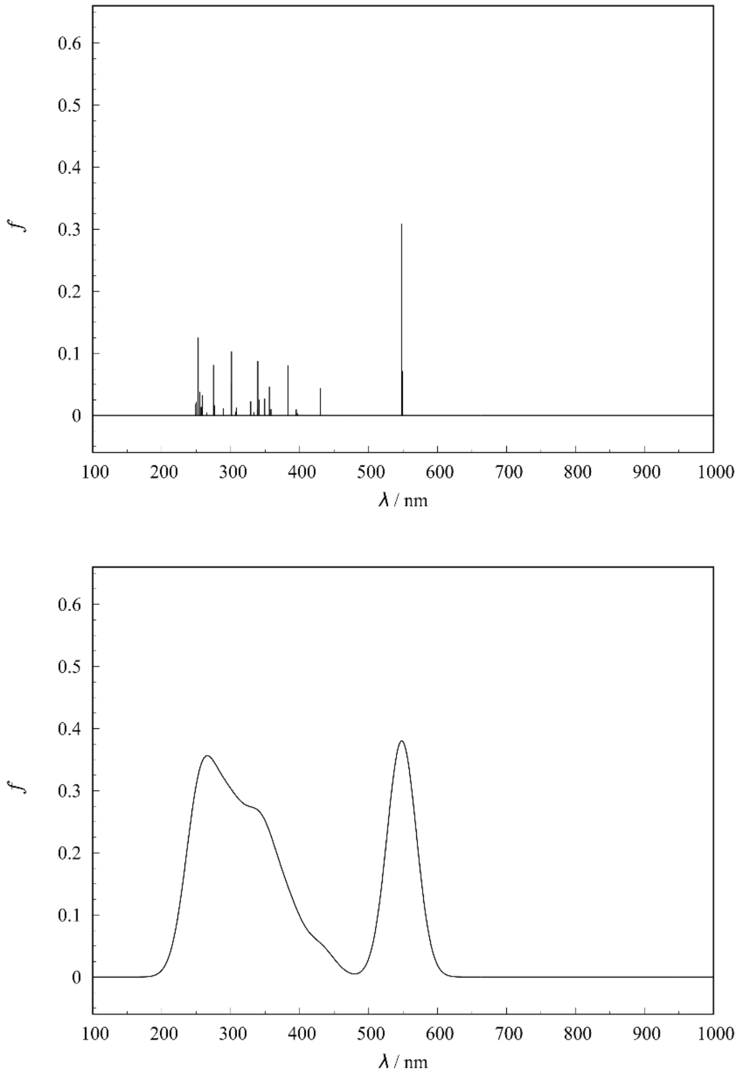

Figure F11. UV-visible spectrum (lines: top; Gaussian line broadening with a full width at half maximum (fwhm) of 50 cm^{-1}: bottom) of **5** calculated at TD-PCM(thf)/M06-2X/def2-SVP.

Table T9. Wavelength (*λ*), oscillator strength (*f*) and main assignment of the TD-PCM(thf)/M06-2X/def2-SVP results for compound **6**; threshold for printing excitations was chosen to be $f \geq 0.05$.

**state no.**	***λ* / nm**	***f***	**Assignment**
2	581.9	0.1939	H−2 → L (*c* = 0.4609)
7	403.6	0.0813	H−7 → L (*c* = 0.5+98)
9	391.5	0.0504	H−4 → L (*c* = 0.5606)
16	366.9	0.0766	H−14 → L (*c* = 0.6173)
21	355.2	0.0530	H−8 → L+1 (*c* = 0.3701)
26	345.3	0.1061	H → L+2 (*c* = 0.6157)
36	298.2	0.1162	H → L+4 (*c* = 0.2518)
41	276.5	0.0729	H−2 → L+2 (*c* = 0.2070)
43	268.7	0.0609	H−10 → L+3 (*c* = 0.0725)

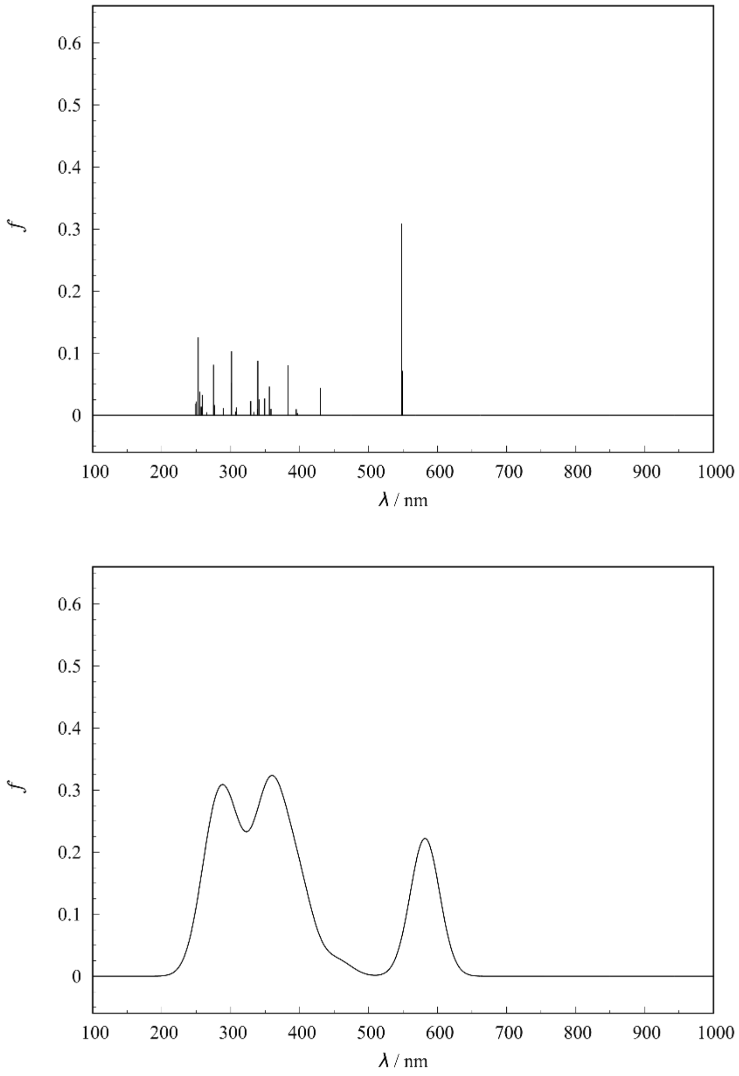

Figure F12. UV-visible spectrum (lines: top; Gaussian line broadening with a fwhm of 50 cm^{-1}: bottom) of **6** calculated at TD-PCM(thf)/M06-2X/def2-SVP.

Table T10. Calculated g-factor and hyperfine coupling constants (A in G), Löwdin and Mulliken (in parenthesis) spin densities for the diarsene radical cations **3** and **4** calculated at the TPSS/def2-TZVP//M06-2X/def2-SVP level of theory with decontracted and ZORA-recontracted basis sets, respectively.

	**3**		**4**	
	decon-def2-TZVP	ZORA-def2-TZVP	decon-def2-TZVP	ZORA-def2-TZVP
g-factor	1.9996874	2.0001811	2.0040740	2.0040773
A_{iso}(As)	−3.2626	−6.9947	−2.4134	−8.0008
$A_{iso}(N_{carb})$	2.7653	2.7002	1.7170	1.6093
$A_{iso}(N_{carb})$	3.2867	3.2556	2.3119	2.2581
$A_{iso}(H_{Ph\text{-}ortho})$	−0.5209	−0.4965	−0.1693	−0.1262
$A_{iso}(H_{Ph\text{-}ortho})$	−0.8092	−0.8034	−0.7381	−0.7219
$A_{iso}(H_{Ph\text{-}para})$	−0.4576	−0.4839	−0.5388	−0.5622
$A_{iso}(H_{carb})$	−1.3740	−1.4856	2.6411	2.6898
$A_{iso}(H_{carb})$	−1.7315	−1.8481	6.9413	6.9712
$A_{iso}(H_{carb})$	–	–	1.2638	1.2818
$A_{iso}(H_{carb})$	–	–	6.9727	7.0129
ρ_{spin}(As)	0.11(0.12)	0.12(0.12)	0.15(0.16)	0.15(0.16)
$\rho_{spin}(C_{vinylic})$	0.16(0.20)	0.16(0.20)	0.14(0.15)	0.14(0.15)
$\rho_{spin}(C_{carb\text{-}ipso})$	0.03(0.02)	0.01(0.03)	0.06(0.07)	0.06(0.07)
$\rho_{spin}(N_{carb})$	0.04(0.05)	0.04(0.05)	0.04(0.05)	0.04(0.05)
$\rho_{spin}(N_{carb})$	0.04(0.05)	0.04(0.05)	0.03(0.05)	0.04(0.05)
$\rho_{spin}(C_{carb})$	0.02(0.02)	0.02(0.02)	0.04(0.00)	0.00(0.00)
$\rho_{spin}(C_{carb})$	0.02(0.02)	0.02(0.03)	0.05(0.00)	0.00(0.00)
$\rho_{spin}(C_{Ph\text{-}ipso})$	0.01(−0.02)	0.01(−0.02)	0.01(−0.02)	0.01(−0.02)
$\rho_{spin}(C_{Ph\text{-}ortho})$	0.01(0.01)	0.01(0.01)	0.01(0.01)	0.01(0.01)
$\rho_{spin}(C_{Ph\text{-}ortho})$	0.01(0.01)	0.01(0.01)	0.01(0.01)	0.01(0.01)
$\rho_{spin}(C_{Ph\text{-}para})$	0.01(0.01)	0.01(0.01)	0.01(0.01)	0.00(0.01)

Table T11. Least-squares-optimized spin Hamiltonian parameters and linewidths used in the simulations of the EPR spectra of **3** and **4**, using a minimalistic model that included the g values and the hyperfine couplings of both As atoms.

	**3**	**4**
g_x	2.0026	2.0035
g_y	1.99882	2.00314
g_z	2.00942	2.00628
As1 Ax [MHz]	-103.9	-71.6
As1 Ay [MHz]	-40.1	-53.2
As1 Az [MHz]	97.0	166.8
As2 Ax [MHz]	-75.7	-33.2
As2 Ay [MHz]	-39.7	-66.3
As2 Az [MHz]	71.3	166.5
H1 Ax [MHz]	14.5	10.8
H1 Ay [MHz]	-3.9	10.6
H1 Az [MHz]	-20.5	-20.0
H2 Ax [MHz]	21.3	15.1
H2 Ay [MHz]	12.1	14.7
H2 Az [MHz]	-5.7	-29.2
Linewidth		
Lx [MHz]	33.7	81
Ly [MHz]	64.3	74
Lz [MHz]	104.5	113

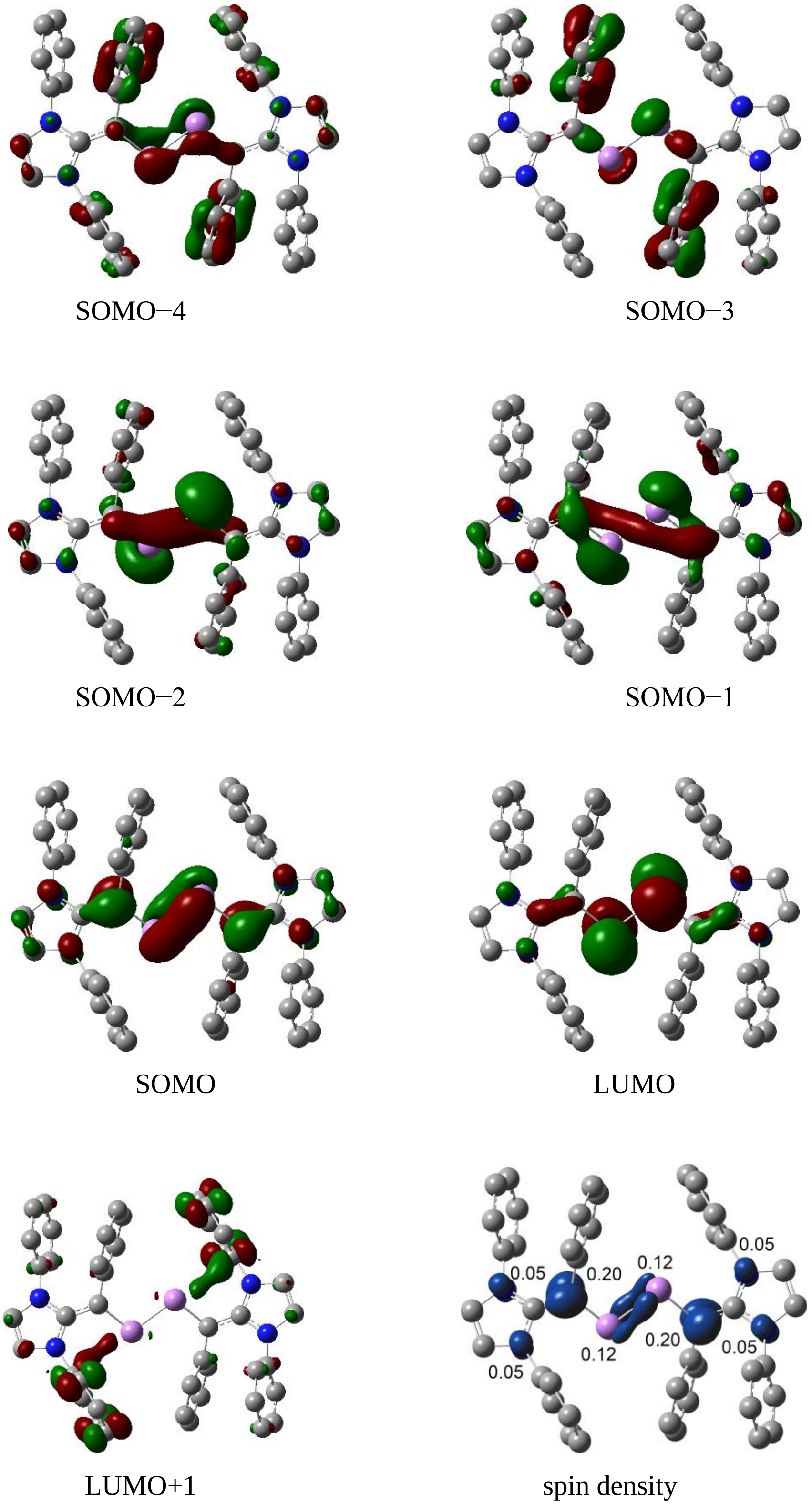

Figure F13. Selected molecular orbitals (from SOMO−4 to LUMO+1) and spin density plot of compound **3**, calculated at M06-2X/def2-TZVPP//def2-SVP. The isovalue was arbitrarily chosen to be 0.04 for molecular orbitals and 0.004 for spin density. Hydrogen atoms as well as *iso*-propyl groups were omitted for clarity.

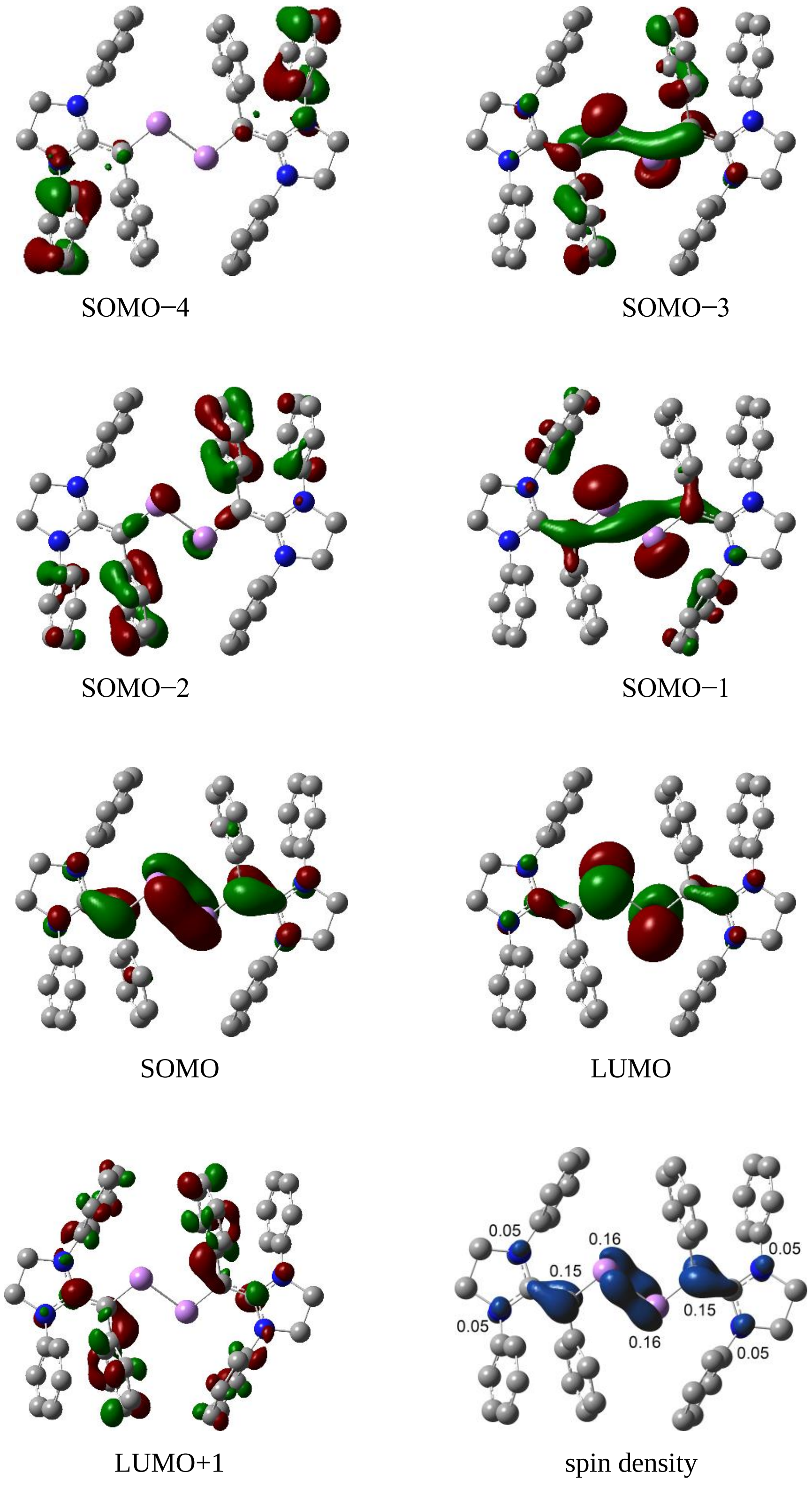

Figure F14. Selected molecular orbitals (from SOMO−4 to LUMO+1) and spin density plot of compound **4**, calculated at M06-2X/def2-TZVPP//def2-SVP. The isovalue was arbitrarily chosen to be 0.04 for molecular orbitals and 0.004 for spin density. Hydrogen atoms as well as *iso*-propyl groups were omitted for clarity.

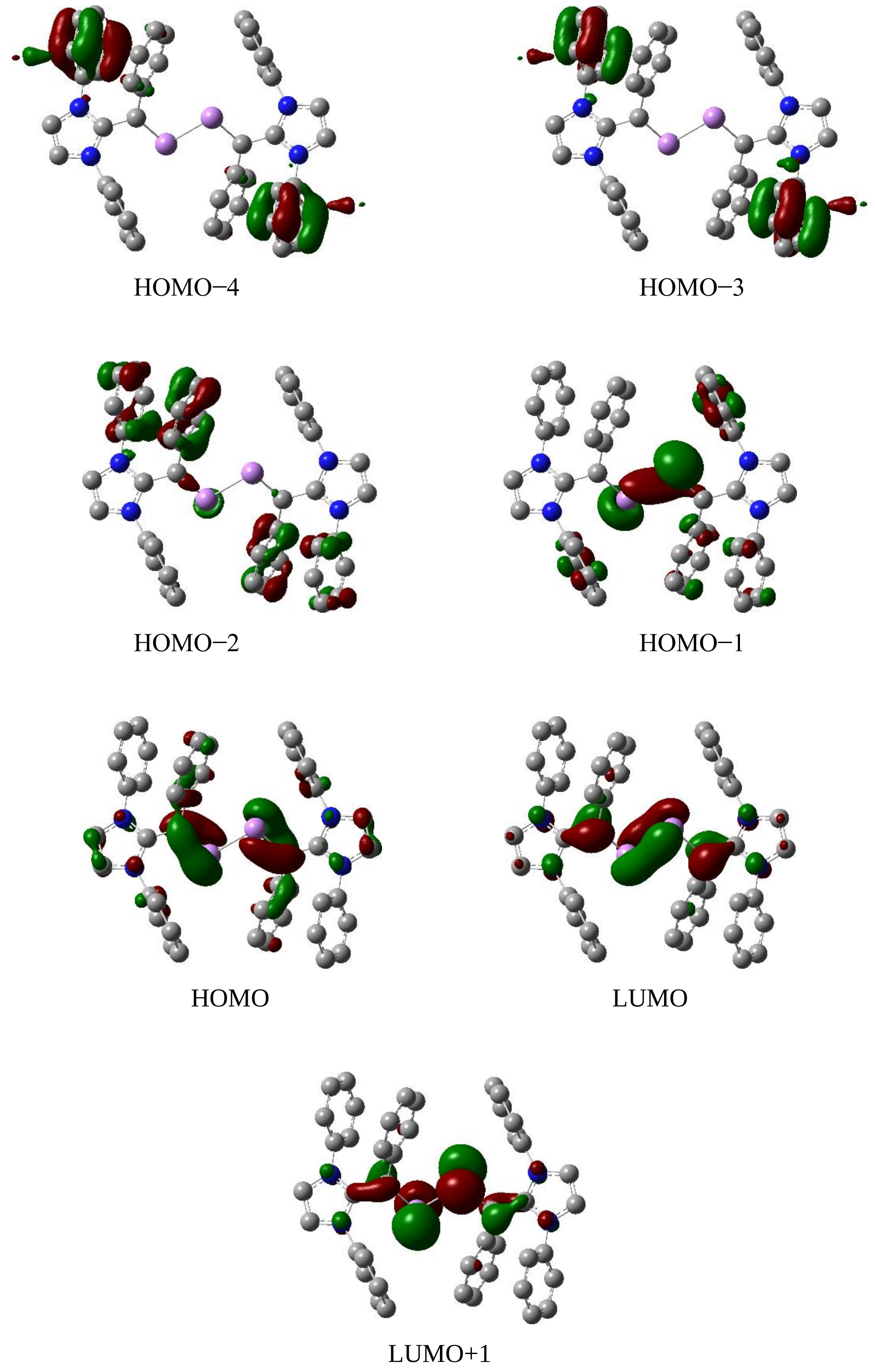

Figure F15. Selected molecular orbitals (from HOMO−4 to LUMO+1) of compound **5**, calculated at M06-2X/def2-TZVPP//def2-SVP. The isovalue was arbitrarily chosen to be 0.04. Hydrogen atoms as well as *iso*-propyl groups were omitted for clarity.

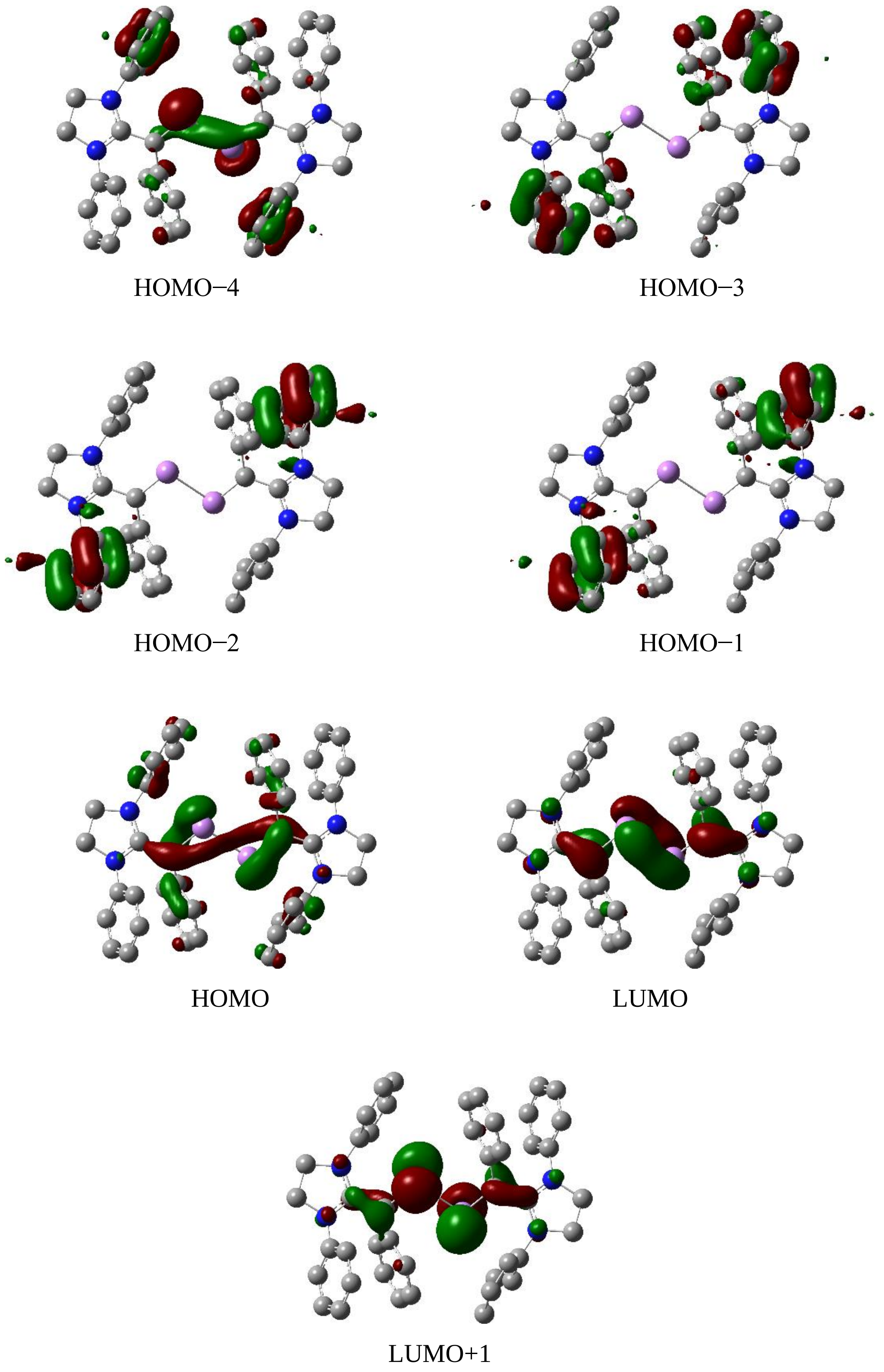

Figure F16. Selected molecular orbitals (from HOMO−4 to LUMO+1) of compound **6**, calculated at M06-2X/def2-TZVPP//def2-SVP. The isovalue was arbitrarily chosen to be 0.04. Hydrogen atoms as well as *iso*-propyl groups were omitted for clarity.

Table T12. Carstesian coordinates (in Å) of the molecular structures of **3, 4**, **5** and **6** optimized at M06-2X/def2-SVP level of theory.

**3**	ν_{min} = 8 cm^{-1}		E = -7327.3842962
As	-0.685535	-0.873790	-0.365745
N	-4.765204	-0.584651	-0.180162
N	-3.559278	-2.366098	0.177009
C	-3.487737	-1.004588	0.074486
C	-5.610774	-1.676992	-0.253295
H	-6.661868	-1.567135	-0.495840
C	-4.863976	-2.782383	-0.021332
H	-5.135404	-3.831003	0.040771
C	-2.331297	-0.175142	0.173536
C	-2.483038	1.207199	0.708395
C	-2.295047	2.335033	-0.106041
H	-2.087761	2.196598	-1.169100
C	-2.379562	3.618390	0.425232
H	-2.248901	4.480573	-0.230308
C	-2.621695	3.801761	1.787918
H	-2.675423	4.808095	2.206830
C	-2.790666	2.690262	2.611724
H	-2.972574	2.821952	3.679820
C	-2.735398	1.403976	2.073565
H	-2.862259	0.534408	2.721802
C	-5.103080	0.721430	-0.688533
C	-5.750077	1.647229	0.150093
C	-6.003163	2.914624	-0.379738
H	-6.489088	3.668242	0.239741
C	-5.639430	3.234408	-1.685791
H	-5.845496	4.232276	-2.075635
C	-5.031259	2.285537	-2.498999
H	-4.774146	2.540526	-3.528946
C	-4.751386	1.001952	-2.019168
C	-6.195473	1.282328	1.557165
H	-5.457765	0.567188	1.957764
C	-7.570813	0.598586	1.533864
H	-8.320061	1.274408	1.095327
H	-7.890797	0.351925	2.556440
H	-7.573226	-0.329766	0.949828
C	-6.256469	2.485799	2.499196
H	-6.382760	2.141844	3.535543
H	-7.119258	3.127575	2.265536
H	-5.346348	3.095623	2.441578
C	-4.144959	-0.036352	-2.952019
H	-3.919905	-0.943113	-2.371354
C	-2.830607	0.434843	-3.580070
H	-2.982526	1.324602	-4.209536
H	-2.413504	-0.356673	-4.220151
H	-2.082105	0.674611	-2.810918
C	-5.162357	-0.431378	-4.029390
H	-4.752163	-1.222165	-4.674239
H	-6.095681	-0.800446	-3.580407
H	-5.413418	0.429682	-4.666854
C	-2.511934	-3.284776	0.544179
C	-2.168361	-3.405386	1.898943
C	-1.189462	-4.348381	2.230427
H	-0.900199	-4.473376	3.275634
C	-0.590810	-5.133531	1.252288
H	0.160190	-5.873177	1.534996
C	-0.938264	-4.975176	-0.087058
H	-0.441024	-5.578087	-0.848158
C	-1.899488	-4.040063	-0.473103
C	-2.791030	-2.553407	2.990848
H	-3.492554	-1.845746	2.522393
C	-3.592696	-3.414312	3.972420
H	-4.380593	-3.980013	3.455109
H	-2.940977	-4.136457	4.486567
H	-4.066514	-2.784030	4.738553
C	-1.716283	-1.732522	3.712875
H	-1.014047	-2.387583	4.250076
H	-1.138139	-1.121362	3.002438
H	-2.177249	-1.064170	4.455547
C	-2.242521	-3.843082	-1.940826
H	-2.716484	-2.852504	-2.037834

C	-0.996090	-3.838975	-2.829756
H	-1.268969	-3.542347	-3.852949
H	-0.542980	-4.839860	-2.892527
H	-0.235233	-3.140011	-2.453095
C	-3.244094	-4.900371	-2.421175
H	-3.497325	-4.738132	-3.478873
H	-2.811419	-5.907628	-2.324792
H	-4.177059	-4.879955	-1.841163
As	0.685584	0.873747	0.365669
N	4.765196	0.584687	0.180390
N	3.559304	2.366101	-0.177047
C	3.487770	1.004595	-0.074476
C	5.610741	1.677041	0.253605
H	6.661804	1.567216	0.496294
C	4.863962	2.782412	0.021484
H	5.135394	3.831030	-0.040614
C	2.331341	0.175130	-0.173627
C	2.483117	-1.207183	-0.708547
C	2.295040	-2.335052	0.105831
H	2.087652	-2.196646	1.168873
C	2.379606	-3.618384	-0.425482
H	2.248884	-4.480598	0.230007
C	2.621878	-3.801705	-1.788154
H	2.675634	-4.808028	-2.207088
C	2.790925	-2.690178	-2.611899
H	2.972934	-2.821817	-3.679983
C	2.735605	-1.403907	-2.073695
H	2.862542	-0.534323	-2.721895
C	5.103026	-0.721422	0.688719
C	5.750157	-1.647146	-0.149893
C	6.003190	-2.914577	0.379878
H	6.489219	-3.668134	-0.239593
C	5.639271	-3.234471	1.685853
H	5.845290	-4.232369	2.075646
C	5.030960	-2.285678	2.499046
H	4.773672	-2.540758	3.528926
C	4.751130	-1.002060	2.019275
C	6.195744	-1.282138	-1.556882
H	5.458138	-0.566903	-1.957500
C	7.571134	-0.598508	-1.533351
H	8.320289	-1.274455	-1.094847
H	7.891231	-0.351713	-2.555858
H	7.573564	0.329745	-0.949162
C	6.256745	-2.485515	-2.499032
H	6.383186	-2.141466	-3.535331
H	7.119449	-3.127393	-2.265345
H	5.346560	-3.095261	-2.441582
C	4.144507	0.036155	2.952094
H	3.919342	0.942883	2.371415
C	2.830197	-0.435228	3.580089
H	2.982224	-1.324920	4.209625
H	2.412906	0.356251	4.220091
H	2.081786	-0.675181	2.810904
C	5.161803	0.431330	4.029507
H	4.751449	1.222031	4.674361
H	6.095077	0.800571	3.580563
H	5.412990	-0.429703	4.666957
C	2.511958	3.284766	-0.544246
C	2.168356	3.405297	-1.899012
C	1.189426	4.348243	-2.230527
H	0.900122	4.473154	-3.275734
C	0.590796	5.133459	-1.252423
H	-0.160222	5.873076	-1.535158
C	0.938303	4.975204	0.086917
H	0.441100	5.578179	0.847992
C	1.899542	4.040116	0.473000
C	2.791041	2.553292	-2.990887
H	3.492532	1.845622	-2.522398
C	3.592761	3.414186	-3.972426
H	4.380642	3.979877	-3.455078
H	2.941069	4.136339	-4.486595
H	4.066603	2.783899	-4.738540
C	1.716312	1.732424	-3.712957
H	1.014089	2.387492	-4.250168
H	1.138152	1.121247	-3.002547

H	2.177297	1.064084	-4.455628
C	2.242604	3.843271	1.940736
H	2.716714	2.852769	2.037821
C	0.996171	3.839048	2.829659
H	1.269103	3.542605	3.852892
H	0.542870	4.839855	2.892293
H	0.235445	3.139900	2.453083
C	3.244022	4.900742	2.421005
H	3.497330	4.738593	3.478698
H	2.811180	5.907925	2.324599
H	4.176955	4.880454	1.840938

**4**	ν_{min} = 7 cm^{-1}		*E* = -7329.7766687
As	-0.692857	0.874042	0.355081
N	-4.737467	0.549752	0.061732
N	-3.486826	2.367184	-0.150248
C	-3.469432	1.009476	-0.113993
C	-5.703029	1.651531	-0.025653
C	-4.819314	2.881558	0.181672
C	-2.326377	0.173030	-0.214654
C	-2.494074	-1.213502	-0.730828
C	-2.255461	-2.332611	0.082867
H	-1.983536	-2.184435	1.130350
C	-2.380982	-3.622185	-0.425174
H	-2.210369	-4.476284	0.231468
C	-2.717484	-3.820352	-1.765267
H	-2.805249	-4.831328	-2.166693
C	-2.941311	-2.717977	-2.588351
H	-3.201026	-2.861667	-3.638576
C	-2.845876	-1.425774	-2.071070
H	-3.025947	-0.564850	-2.717507
C	-5.039406	-0.692772	0.712286
C	-5.764298	-1.677318	0.011985
C	-5.977000	-2.904315	0.643659
H	-6.518371	-3.691980	0.118882
C	-5.505669	-3.138681	1.932572
H	-5.677437	-4.105924	2.406992
C	-4.839932	-2.134114	2.625004
H	-4.508425	-2.315232	3.649260
C	-4.600100	-0.887968	2.036815
C	-6.356971	-1.396819	-1.357451
H	-5.708106	-0.649920	-1.844835
C	-7.767918	-0.805201	-1.205658
H	-8.436133	-1.551342	-0.750088
H	-8.182279	-0.532478	-2.187390
H	-7.787407	0.084099	-0.562257
C	-6.425955	-2.630746	-2.258551
H	-6.690094	-2.332304	-3.283186
H	-7.204531	-3.328950	-1.916988
H	-5.469296	-3.167147	-2.285162
C	-3.956821	0.210706	2.871977
H	-3.704836	1.055590	2.216630
C	-2.655755	-0.235673	3.543861
H	-2.839771	-1.011903	4.301638
H	-2.179905	0.616102	4.052434
H	-1.939860	-0.633843	2.809766
C	-4.957328	0.733618	3.911114
H	-4.519715	1.563508	4.485960
H	-5.881483	1.091118	3.433544
H	-5.236146	-0.060691	4.619661
C	-2.455182	3.259420	-0.591034
C	-2.127956	3.318019	-1.958960
C	-1.151217	4.237241	-2.356964
H	-0.878518	4.302185	-3.412161
C	-0.539201	5.081455	-1.438065
H	0.206874	5.803349	-1.774568
C	-0.877556	5.005592	-0.090184
H	-0.382324	5.660560	0.628907
C	-1.828934	4.089763	0.361177
C	-2.774882	2.431960	-3.009825
H	-3.480297	1.752749	-2.507658
C	-3.578464	3.265774	-4.014006
H	-4.345529	3.870469	-3.509118
H	-2.923608	3.953042	-4.569996
H	-4.077809	2.613195	-4.744648

C	-1.723706	1.570080	-3.718519
H	-1.010960	2.196564	-4.275478
H	-1.154654	0.962892	-2.997455
H	-2.202118	0.892892	-4.441949
C	-2.151934	4.004735	1.843619
H	-2.714179	3.070287	2.005047
C	-0.889136	3.928903	2.707899
H	-1.162717	3.742109	3.756832
H	-0.328072	4.875168	2.681313
H	-0.215931	3.126773	2.372419
C	-3.028345	5.186570	2.278852
H	-3.321007	5.086507	3.334342
H	-2.472597	6.130341	2.170519
H	-3.940326	5.272885	1.670981
As	0.692833	-0.874033	-0.354971
N	4.737445	-0.549757	-0.061866
N	3.486819	-2.367200	0.150017
C	3.469425	-1.009496	0.113925
C	5.703013	-1.651533	0.025349
C	4.819282	-2.881535	-0.182055
C	2.326368	-0.173065	0.214781
C	2.494145	1.213392	0.731127
C	2.255370	2.332630	-0.082343
H	1.983198	2.184620	-1.129785
C	2.381017	3.622124	0.425866
H	2.210270	4.476321	-0.230615
C	2.717803	3.820088	1.765919
H	2.805679	4.831002	2.167475
C	2.941789	2.717583	2.588788
H	3.201718	2.861112	3.638982
C	2.846235	1.425461	2.071328
H	3.026428	0.564438	2.717599
C	5.039340	0.692908	-0.712156
C	5.764296	1.677276	-0.011672
C	5.976986	2.904413	-0.643073
H	6.518394	3.691949	-0.118141
C	5.505587	3.139082	-1.931907
H	5.677363	4.106423	-2.406123
C	4.839782	2.134691	-2.624529
H	4.508238	2.316048	-3.648731
C	4.599956	0.888415	-2.036615
C	6.357039	1.396428	1.357660
H	5.708138	0.649491	1.844935
C	7.767919	0.804699	1.205629
H	8.436169	1.550878	0.750176
H	8.182328	0.531715	2.187268
H	7.787272	-0.084459	0.562027
C	6.426223	2.630147	2.259031
H	6.690441	2.331447	3.283569
H	7.204823	3.328360	1.917544
H	5.469615	3.166629	2.285871
C	3.956610	-0.210061	-2.871992
H	3.704637	-1.055083	-2.216827
C	2.655511	0.236475	-3.543703
H	2.839473	1.012834	-4.301360
H	2.179607	-0.615203	-4.052391
H	1.939671	0.634522	-2.809486
C	4.957062	-0.732752	-3.911297
H	4.519413	-1.562490	-4.486336
H	5.881223	-1.090391	-3.433843
H	5.235877	0.061730	-4.619651
C	2.455183	-3.259503	0.590683
C	2.128013	-3.318320	1.958615
C	1.151333	-4.237643	2.356524
H	0.878687	-4.302764	3.411725
C	0.539310	-5.081743	1.437526
H	-0.206711	-5.803726	1.773955
C	0.877588	-5.005650	0.089639
H	0.382337	-5.660516	-0.629531
C	1.828910	-4.089714	-0.361628
C	2.774930	-2.432356	3.009565
H	3.480367	-1.753124	2.507455
C	3.578475	-3.266239	4.013715
H	4.345520	-3.870944	3.508810
H	2.923598	-3.953499	4.569690

H	4.077839	-2.613700	4.744379
C	1.723738	-1.570515	3.718286
H	1.010979	-2.197047	4.275174
H	1.154703	-0.963281	2.997250
H	2.202132	-0.893377	4.441774
C	2.151820	-4.004461	-1.844078
H	2.714027	-3.069978	-2.005415
C	0.888966	-3.928558	-2.708274
H	1.162479	-3.741620	-3.757199
H	0.327941	-4.874847	-2.681775
H	0.215750	-3.126497	-2.372653
C	3.028240	-5.186210	-2.279527
H	3.320821	-5.085993	-3.335025
H	2.472540	-6.130020	-2.171282
H	3.940270	-5.272568	-1.671736
H	-5.076684	3.715111	-0.484259
H	-4.842491	3.243224	1.223434
H	-6.473453	1.541475	0.747434
H	-6.190978	1.655945	-1.013693
H	6.473399	-1.541397	-0.747764
H	6.191016	-1.656055	1.013364
H	5.076703	-3.715166	0.483758
H	4.842388	-3.243078	-1.223862

**5**	ν_{min} = 6 cm^{-1}		E = -7327.1127422
As	-0.750987	-0.827558	-0.494788
N	-4.716247	-0.492951	-0.364884
N	-3.625687	-2.305757	0.137835
C	-3.504552	-0.963453	0.009428
C	-5.600422	-1.539070	-0.470232
H	-6.624475	-1.393363	-0.797124
C	-4.922065	-2.672978	-0.138180
H	-5.244982	-3.707206	-0.070706
C	-2.300514	-0.152121	0.185621
C	-2.458703	1.152596	0.874825
C	-2.115793	2.350190	0.225240
H	-1.787331	2.321079	-0.816460
C	-2.232557	3.569300	0.887695
H	-1.986651	4.492016	0.360925
C	-2.659969	3.607385	2.215217
H	-2.739503	4.561106	2.739059
C	-2.995130	2.421707	2.870556
H	-3.329444	2.446084	3.908742
C	-2.922233	1.203659	2.197821
H	-3.195119	0.280642	2.712578
C	-4.964867	0.859942	-0.819810
C	-5.701559	1.733235	-0.000507
C	-5.849790	3.045764	-0.457422
H	-6.404188	3.763499	0.147256
C	-5.306182	3.451258	-1.673255
H	-5.440892	4.480390	-2.008825
C	-4.613084	2.548497	-2.472824
H	-4.221334	2.874526	-3.437291
C	-4.424985	1.224489	-2.065661
C	-6.365117	1.280691	1.290209
H	-5.762447	0.454576	1.705673
C	-7.783598	0.759287	1.009216
H	-8.401430	1.565420	0.587412
H	-8.257122	0.420074	1.941130
H	-7.802903	-0.074621	0.296414
C	-6.447440	2.387746	2.343840
H	-6.743792	1.960262	3.311732
H	-7.211802	3.131669	2.076361
H	-5.490386	2.909107	2.467725
C	-3.745732	0.230943	-2.997398
H	-3.437358	-0.649823	-2.414362
C	-2.481961	0.793487	-3.651570
H	-2.717379	1.594190	-4.367485
H	-1.963207	0.000649	-4.209768
H	-1.783563	1.198720	-2.902952
C	-4.745269	-0.254615	-4.055814
H	-4.278482	-1.000891	-4.714943
H	-5.632080	-0.709929	-3.591567
H	-5.087633	0.585147	-4.678302
C	-2.597969	-3.265827	0.483970

C	-2.263777	-3.440156	1.834985
C	-1.295495	-4.406367	2.129550
H	-1.012615	-4.578995	3.169502
C	-0.706645	-5.162405	1.122075
H	0.030479	-5.925230	1.378024
C	-1.057961	-4.956890	-0.210139
H	-0.585576	-5.553950	-0.991423
C	-2.007680	-3.996857	-0.564771
C	-2.888010	-2.634294	2.959986
H	-3.605985	-1.922880	2.520601
C	-3.672122	-3.538254	3.917690
H	-4.451499	-4.102988	3.387444
H	-3.007153	-4.262522	4.410428
H	-4.153924	-2.938448	4.702353
C	-1.817515	-1.827298	3.704476
H	-1.100909	-2.495089	4.205076
H	-1.253954	-1.175489	3.018097
H	-2.278162	-1.198629	4.480676
C	-2.376621	-3.783182	-2.024777
H	-2.865704	-2.798006	-2.110128
C	-1.148409	-3.761856	-2.939739
H	-1.445907	-3.462631	-3.954847
H	-0.691163	-4.759079	-3.020142
H	-0.380748	-3.061469	-2.579164
C	-3.374421	-4.849641	-2.495731
H	-3.656202	-4.676057	-3.543791
H	-2.923714	-5.850661	-2.425827
H	-4.292417	-4.856565	-1.892283
As	0.744255	0.834030	0.473696
N	4.708658	0.477180	0.380817
N	3.634339	2.305716	-0.098642
C	3.503785	0.961925	0.002657
C	5.597685	1.515725	0.516277
H	6.617024	1.357295	0.851922
C	4.929714	2.659814	0.198688
H	5.259475	3.693125	0.154203
C	2.296959	0.161061	-0.200494
C	2.454433	-1.134116	-0.908985
C	2.113585	-2.341269	-0.276572
H	1.787659	-2.328033	0.766230
C	2.228585	-3.550096	-0.957828
H	1.984106	-4.480750	-0.444450
C	2.651616	-3.567918	-2.287243
H	2.729334	-4.513548	-2.825798
C	2.984303	-2.372394	-2.925517
H	3.315202	-2.380824	-3.965046
C	2.913134	-1.164612	-2.234087
H	3.182922	-0.233516	-2.735747
C	4.946359	-0.885321	0.811951
C	5.683116	-1.747189	-0.019339
C	5.823552	-3.068178	0.414892
H	6.377819	-3.777504	-0.199742
C	5.272342	-3.492843	1.620817
H	5.401480	-4.528241	1.938869
C	4.578548	-2.601631	2.432539
H	4.180303	-2.942913	3.389117
C	4.397811	-1.269849	2.047818
C	6.353434	-1.274811	-1.299428
H	5.754808	-0.439940	-1.703033
C	7.772336	-0.762496	-1.004439
H	8.385798	-1.576865	-0.592174
H	8.250916	-0.410904	-1.929152
H	7.791082	0.060856	-0.279460
C	6.435999	-2.364117	-2.371359
H	6.738043	-1.921340	-3.330569
H	7.196384	-3.115442	-2.113359
H	5.477381	-2.879360	-2.508185
C	3.714774	-0.291559	2.992805
H	3.425885	0.606809	2.426841
C	2.434234	-0.856339	3.611879
H	2.649276	-1.679380	4.308714
H	1.915746	-0.072979	4.183693
H	1.742854	-1.232197	2.841741
C	4.701887	0.156132	4.079095
H	4.232670	0.891283	4.748864

H	5.601138	0.612382	3.640538
H	5.024627	-0.702477	4.686177
C	2.618074	3.277515	-0.445370
C	2.313743	3.480568	-1.799372
C	1.356186	4.457121	-2.095027
H	1.096756	4.652511	-3.137043
C	0.748511	5.194678	-1.085132
H	0.019957	5.965649	-1.341272
C	1.070267	4.960834	0.249940
H	0.583297	5.543929	1.032782
C	2.009213	3.990516	0.605165
C	2.961746	2.697117	-2.926775
H	3.655132	1.962444	-2.486098
C	3.788537	3.617135	-3.831984
H	4.559561	4.153220	-3.261234
H	3.149366	4.366521	-4.321322
H	4.285297	3.034162	-4.620013
C	1.905434	1.927292	-3.728114
H	1.217297	2.618310	-4.236843
H	1.308210	1.268531	-3.078055
H	2.383348	1.310585	-4.503453
C	2.350566	3.748625	2.067439
H	2.827474	2.756729	2.144005
C	1.107662	3.726529	2.962135
H	1.385262	3.406204	3.976432
H	0.661275	4.727775	3.052855
H	0.337446	3.041995	2.577464
C	3.352221	4.795829	2.572388
H	3.614414	4.601870	3.621967
H	2.913828	5.802874	2.511500
H	4.280248	4.802057	1.984535

**6**	ν_{min} = 3 cm^{-1}		E = -7329.4924223
As	-0.757619	0.855378	0.427337
N	-4.689405	0.467596	0.215491
N	-3.566247	2.313344	-0.240423
C	-3.502121	0.985816	-0.106405
C	-5.682168	1.537235	0.410514
C	-4.959185	2.772841	-0.136046
C	-2.289482	0.168484	-0.273621
C	-2.443945	-1.134613	-0.965244
C	-2.067021	-2.331473	-0.331707
H	-1.725278	-2.308041	0.706400
C	-2.170129	-3.547802	-1.001373
H	-1.895457	-4.468291	-0.485564
C	-2.623429	-3.586067	-2.319897
H	-2.693742	-4.537534	-2.848929
C	-3.003457	-2.403890	-2.956462
H	-3.365241	-2.428250	-3.985361
C	-2.940667	-1.189802	-2.276463
H	-3.252330	-0.272816	-2.778751
C	-4.877455	-0.845844	0.777927
C	-5.664447	-1.777387	0.073498
C	-5.770902	-3.064412	0.605880
H	-6.361670	-3.814731	0.079478
C	-5.143177	-3.401602	1.801382
H	-5.241384	-4.412217	2.199955
C	-4.420361	-2.445247	2.506015
H	-3.972223	-2.711055	3.464790
C	-4.276650	-1.142500	2.018357
C	-6.448362	-1.398441	-1.171001
H	-5.960849	-0.515629	-1.618449
C	-7.889932	-1.029589	-0.780016
H	-8.410706	-1.920302	-0.398755
H	-8.446744	-0.661648	-1.653590
H	-7.937625	-0.270098	0.012790
C	-6.482529	-2.503799	-2.229019
H	-6.920008	-2.117474	-3.160215
H	-7.112763	-3.344581	-1.905424
H	-5.479512	-2.891082	-2.445987
C	-3.571692	-0.106339	2.884575
H	-3.353224	0.786300	2.278274
C	-2.237344	-0.610481	3.443464
H	-2.391935	-1.384714	4.209230
H	-1.686745	0.214047	3.920589

H	-1.599202	-1.041783	2.656496
C	-4.493633	0.339631	4.028579
H	-4.008852	1.119189	4.634352
H	-5.448194	0.734796	3.652769
H	-4.727030	-0.508510	4.688798
C	-2.524368	3.247376	-0.574073
C	-2.084618	3.356280	-1.905957
C	-1.088042	4.297865	-2.182678
H	-0.726150	4.407709	-3.206717
C	-0.574981	5.116299	-1.182070
H	0.184910	5.860633	-1.426029
C	-1.041828	5.000996	0.124368
H	-0.640416	5.654728	0.901073
C	-2.020193	4.062902	0.460352
C	-2.642636	2.510983	-3.036487
H	-3.404891	1.835544	-2.615772
C	-3.340012	3.386298	-4.085035
H	-4.131037	4.001748	-3.634023
H	-2.624173	4.065570	-4.570371
H	-3.791236	2.761306	-4.868371
C	-1.546687	1.651211	-3.676883
H	-0.775060	2.284051	-4.140044
H	-1.056583	1.001763	-2.934454
H	-1.966900	1.012585	-4.467745
C	-2.509053	3.971919	1.897556
H	-3.162326	3.087558	1.981724
C	-1.354539	3.783789	2.888869
H	-1.750897	3.628634	3.902727
H	-0.712546	4.676551	2.923423
H	-0.721313	2.924158	2.624997
C	-3.331804	5.212968	2.270506
H	-3.738840	5.115037	3.286739
H	-2.700359	6.113391	2.247371
H	-4.167362	5.385240	1.576789
As	0.760845	-0.852471	-0.439413
N	4.692908	-0.475192	-0.209737
N	3.561782	-2.312849	0.259194
C	3.502445	-0.986600	0.110913
C	5.683396	-1.549806	-0.388498
C	4.953929	-2.777439	0.167511
C	2.291252	-0.164335	0.264001
C	2.445510	1.143623	0.945782
C	2.069081	2.335580	0.302676
H	1.728653	2.303948	-0.735644
C	2.171390	3.557107	0.963022
H	1.896949	4.473538	0.439963
C	2.623733	3.605591	2.281495
H	2.693619	4.561129	2.803207
C	3.003554	2.428398	2.927494
H	3.364613	2.460828	3.956427
C	2.941436	1.209119	2.256917
H	3.253045	0.296142	2.766506
C	4.887139	0.832232	-0.783987
C	5.673210	1.768572	-0.084903
C	5.784619	3.050393	-0.628679
H	6.374808	3.804312	-0.106797
C	5.162498	3.377838	-1.829809
H	5.264328	4.384562	-2.237212
C	4.440699	2.416495	-2.528685
H	3.997205	2.674379	-3.491765
C	4.292273	1.118567	-2.029710
C	6.451334	1.399336	1.166120
H	5.959564	0.522296	1.620213
C	7.893217	1.022429	0.784032
H	8.418083	1.907890	0.396217
H	8.445692	0.661160	1.663130
H	7.941484	0.255294	-0.001348
C	6.484957	2.514737	2.213547
H	6.917500	2.136078	3.150184
H	7.119187	3.350307	1.884313
H	5.482365	2.907396	2.422719
C	3.589412	0.075718	-2.889612
H	3.365710	-0.810688	-2.276030
C	2.259239	0.577452	-3.460427
H	2.419479	1.343990	-4.232677

H	1.709389	-0.250402	-3.932503
H	1.618279	1.017626	-2.680750
C	4.516508	-0.383204	-4.024310
H	4.033243	-1.167430	-4.625243
H	5.468161	-0.776817	-3.639593
H	4.755306	0.458097	-4.691307
C	2.513994	-3.239526	0.594809
C	2.062370	-3.331603	1.924020
C	1.057692	-4.264090	2.202196
H	0.686258	-4.360506	3.224180
C	0.548416	-5.090419	1.206073
H	-0.218199	-5.827375	1.451356
C	1.027824	-4.992569	-0.097205
H	0.630045	-5.652920	-0.870177
C	2.014293	-4.063590	-0.434909
C	2.616100	-2.477750	3.050225
H	3.386066	-1.811625	2.628784
C	3.299413	-3.346289	4.113599
H	4.090346	-3.971156	3.675578
H	2.575454	-4.016123	4.600004
H	3.747991	-2.715884	4.894114
C	1.520475	-1.604517	3.672638
H	0.741129	-2.227652	4.136113
H	1.040242	-0.959838	2.919614
H	1.938318	-0.960138	4.460087
C	2.515903	-3.991070	-1.868763
H	3.175179	-3.111603	-1.956884
C	1.371026	-3.806514	-2.871807
H	1.776779	-3.666230	-3.884106
H	0.722747	-4.694923	-2.901084
H	0.742010	-2.939241	-2.623389
C	3.334273	-5.240992	-2.221043
H	3.750751	-5.156584	-3.234652
H	2.697239	-6.137352	-2.193719
H	4.162651	-5.410573	-1.518095
H	-5.309826	3.065468	-1.138368
H	-5.024139	3.646607	0.523847
H	-5.912931	1.612833	1.484451
H	-6.607135	1.311775	-0.131861
H	5.919226	-1.637417	-1.460428
H	6.606346	-1.321454	0.156080
H	5.298310	-3.060017	1.174873
H	5.020096	-3.658533	-0.482444

References

1. M. K. Sharma, S. Blomeyer , B. Neumann, H.-G. Stammler, R. S. Ghadwal, *Chem. Eur. J.* **2019,** *25*, 8249–8253.
2. G. R. Fulmer, A. J. M. Miller, N. H. Sherden, H. E. Gottlieb, A. Nudelman, B. M. Stoltz, J. E. Bercaw, K. I. Goldberg, *Organometallics* **2010**, *29*, 2176–2179.
3. O. V. Dolomanov, L. J. Bourhis, R. J. Gildea, J. A. K. Howard, H. Puschmann, *J. Appl. Cryst.* **2009**, *42*, 339–341.
4. G. M. Sheldrick, *Acta Cryst.* **2008**, *A64*, 112–122.
5. G. M. Sheldrick, *Acta Cryst.* **2015**, *C71*, 3–8.
6. M. J. Frisch, G. W. Trucks, H. B. Schlegel, G. E. Scuseria, M. A. Robb, J. R. Cheeseman, G. Scalmani, V. Barone, B. Mennucci, G. A. Petersson, H. Nakatsuji, M. Caricato, X. Li, H. P. Hratchian, A. F. Izmaylov, J. Bloino, G. Zheng, J. L. Sonnenberg, M. Hada, M. Ehara, K. Toyota, R. Fukuda, J. Hasegawa, M. Ishida, T. Nakajima, Y. Honda, O. Kitao, H. Nakai, T. Vreven, J. J. A. Montgomery, J. E. Peralta, F. Ogliaro, M. Bearpark, J. J. Heyd, E. Brothers, K. N. Kudin, V. N. Staroverov, R. Kobayashi, J. Normand, K. Raghavachari, A. Rendell, J. C. Burant, S. S. Iyengar, J. Tomasi, M. Cossi, N. Rega, J. M. Millam, M. Klene, J. E. Knox, J. B. Cross, V. Bakken, C. Adamo, J. Jaramillo, R. Gomperts, R. E. Stratmann, O. Yazyev, A. J. Austin, R. Cammi, C. Pomelli, J. W. Ochterski, R. L. Martin, K. Morokuma, V. G. Zakrzewski, G. A. Voth, P. Salvador, J. J. Dannenberg, S. Dapprich, A. D. Daniels, O. Farkas, J. B. Foresman, J. V. Ortiz, J. Cioslowski, D. J. Fox, Gaussian 16, Revision A.03. Gaussian, Inc., Wallingford CT, 2016.
7. Y. Zhao, D. G. Truhlar, *Theor. Chem. Acc.* **2008**, *120*, 215–241.
8. F. Weigend, R. Ahlrichs, *Phys. Chem. Chem. Phys.* **2005**, *7*, 3297–3305.
9. J. W. McIver, Komornic.A, *J. Am. Chem. Soc.* **1972**, *94*, 2625–2633.
10. K. B. Wiberg, *Tetrahedron* **1968**, *24*, 1083–1096.
11. (a) A. E. Reed, F. Weinhold, *J. Chem. Phys.* **1985**, *83*, 1736–1740; (b) A. E. Reed, R. B. Weinstock, F. Weinhold, *J. Chem. Phys.* **1985**, *83*, 735–746.
12. E. D. Glendening, A. E. Reed, J. E. Carpenter, F. Weinhold, NBO Version 3.1.
13. F. Neese, *WIREs Comput. Mol. Sci.* **2017**, *8*, e1327.
14. V. Barone, M. Cossi, *J. Phys. Chem. A* **1998**, *102*, 1995.
15. M. Isegawa, F. Neese, D. A. Pantazis, *J. Chem. Theory Comput.* **2016**, *12*, 2272–2284.
16. D. G. Truhlar, C. J. Cramer, A. Lewis, J. A. Bumpus, *J. Chem. Edu.* **2004**, *81*, 596–604.
